# Prior *Trichinella spiralis* infection protects against *Schistosoma mansoni* induced hepatic fibrosis

**DOI:** 10.3389/fvets.2024.1443267

**Published:** 2024-10-08

**Authors:** Asmaa M. El-kady, Sarah A. Altwaim, Majed H. Wakid, Alaa S. Banjar, Khalil Mohammed, Mashael S. Alfaifi, Hayam Elshazly, Wafa Abdullah I. Al-Megrin, Eman Abdullah Alshehri, Eman Sayed, Hatem A. Elshabrawy

**Affiliations:** ^1^Department of Medical Parasitology, Faculty of Medicine, South Valley University, Qena, Egypt; ^2^Department of Clinical Microbiology and Immunology, Faculty of Medicine, King Abdulaziz University, Jeddah, Saudi Arabia; ^3^Special Infectious Agents Unit, King Fahd Medical Research Center, Jeddah, Saudi Arabia; ^4^Department of Medical Laboratory Sciences, Faculty of Applied Medical Sciences, King Abdulaziz University, Jeddah, Saudi Arabia; ^5^Center of Innovation in Personalized Medicine (CIPM), King Abdulaziz University, Jeddah, Saudi Arabia; ^6^Department of Epidemiology and Medical Statistics, Faculty of Public Health and Health Informatics, Umm Al-Qura University, Mecca, Saudi Arabia; ^7^Department of Biology, Faculty of Sciences-Scientific Departments, Qassim University, Buraidah, Qassim, Saudi Arabia; ^8^Department of Zoology, Faculty of Science, Beni-Suef University, Beni Suef, Egypt; ^9^Department of Biology, College of Science, Princess Nourah bint Abdulrahman University, Riyadh, Saudi Arabia; ^10^Department of Zoology, College of Science, King Saud University, Riyadh, Saudi Arabia; ^11^Department of Parasitology, Faculty of Veterinary Medicine, South Valley University, Qena, Egypt; ^12^Department of Molecular and Cellular Biology, College of Osteopathic Medicine, Sam Houston State University, Conroe, TX, United States

**Keywords:** *Schistosoma mansoni*, *Trichinella spiralis*, murine model, hepatic fibrosis, fibrosis markers

## Abstract

**Background:**

Schistosomiasis affects approximately 250 million people worldwide, with 200,000 deaths annually. It has been documented that the granulomatous response to *Schistosoma mansoni* (*S. mansoni*) oviposition is the root cause of progressive liver fibrosis in chronic infection, in 20% of the patients, and can lead to liver cirrhosis and/or liver cancer. The influence of helminths coinfection on schistosomiasis-induced liver pathological alterations remains poorly understood. Therefore, in this study, we investigated the effect of *Trichinella spiralis* (*T. spiralis*) infection on *S. mansoni*-induced hepatic fibrosis.

**Materials and methods:**

Thirty adult male Balb-c mice were divided into three groups. Group 1 was left uninfected; group 2 was infected with *S. mansoni* cercariae and group 3 was orally infected with *T. spiralis* larvae, then 28 days later, this group was infected with *S. mansoni* cercariae. All groups were sacrificed at the end of the 8^th^ week post infection with *S. mansoni to evaluate* the effect of pre-infection with *T. spiralis* on *S. mansoni* induced liver fibrosis was evaluated parasitologically (worm burden and egg count in tissues), biochemically (levels of alanine aminotransferase and aspartate aminotransferase), histopathologically (H&E and MT staining, and immunohistochemical staining for the expression of α-SMA, IL-6, IL-1β, IL-17, IL-23, TNF-α, and TGF-β).

**Results:**

The results in the present study demonstrated marked protective effect of *T. spiralis* against *S. mansoni* induced liver pathology. We demonstrated that pre-infection with *T. spirais* caused marked reduction in the number of *S. mansoni* adult worms (3.17 ± 0.98 vs. 18 ± 2.16, *P* = 0.114) and egg count in both the intestine (207.2 ± 64.3 vs. 8,619.43 ± 727.52, *P* = 0.009) and liver tissues (279 ± 87.2 vs. 7,916.86 ± 771.34; *P* = 0.014). Consistently, we found significant reductions in both number (3.4 ± 1.1 vs. 11.8.3 ± 1.22; *P* = 0.007) and size (84 ± 11 vs. 294.3 ± 16.22; *P* = 0.001) of the hepatic granulomas in mice pre-infected with *T. spiralis* larvae compared to those infected with only *S. mansoni*. Furthermore, pre- infection with *T. spiralis* markedly reduced *S. mansoni*- induced hepatic fibrosis, as evidenced by decreased collagen deposition, low expression of α-SMA, and significantly reduced levels of IL-17, IL-1B, IL-6, TGF-B, IL-23, and TNF-α compared to mice infected with *S. mansoni* only.

**Conclusions:**

Our data show that pre-infection with *T. spiralis* effectively protected mice from severe schistosomiasis and liver fibrosis. We believe that our findings support the potential utility of helminths for the preventing and ameliorating severe pathological alterations induced by schistosomiasis.

## 1 Introduction

Although helminths are responsible for causing many diseases in animals and humans (1, 2), it has been noted that the lowest frequency of autoimmune and allergic illnesses is correlated with the highest density of helminth infections (3). The “hygiene hypothesis,” which was developed in response to this observation, contends that helminth infections can both prevent and shield against the development of aberrant adaptive immune responses to normally non-immunogenic foreign or self-antigens, and that living in an exceptionally clean environment predisposes humans to such conditions (4–8). Supporting data from animal models of inflammatory bowel illness (9) and experimental allergic encephalomyelitis (10, 11), type 1 diabetes (9, 12, 13), experimental asthma (14), and Graves' thyroiditis (15), has significantly supported this theory. Coinfections with helminths, predictably attenuate proinflammatory responses against other pathogens, typically leading to decreased immunopathology overall, though occasionally at the expense of decreased protection (16–22). The ability of helminths to reduce inflammation through the induction of anti-inflammatory Th2-type cells, T-regulatory cells (Treg), and alternatively activated macrophages (AAM) has been linked to the ameliorating effect of these organisms on disease susceptibility or magnitude (23).

Chronic schistosomiasis is considered one of the most serious helminth diseases known to humanity especially in tropical and subtropical regions (24–28). Approximately 250 million people are affected worldwide, with more than 200,000 deaths annually (29). Schistosomiasis causes more than 1.8 million disability-adjusted life years (DALYs) (30, 31). It is estimated that at least 220 million people need preventive treatment (31–33). The granulomatous response to *Schistosoma* oviposition and subsequent progressive liver fibrosis in chronic infection are the main pathological lesions of intestinal schistosomiasis (34). Liver fibrosis results from massive deposition of extracellular matrix in the periportal space, leading to portal vein occlusion and a number of complications such as portal hypertension, splenomegaly, portacaval shunt, gastrointestinal disorders, and varicose veins (35). Previous reports indicated that approximately 20% of schistosomiasis patients develop liver fibrosis (36), which may be a risk factor for liver cirrhosis and/or liver cancer with high mortality (37).

Keeping in view the recent developments in vaccine designing and nano-medicine to curb the prevalence of helminthic infestation (38, 39), there is need to put more efforts to design control and treatment strategies against schistosomiasis. Previous studies have investigated the effect of pre-infection with some parasites on *S. mansoni*-induced liver pathology with different outcomes. Regarding co-infection of *Schistosoma* and protozoan parasites, the researchers demonstrated that mice pre-infected with either *T. gondii* or *T. brucei* before *S. mansoni* infection were protected against *S. mansoni* induced liver pathology (40, 41). On the other hand, pre-infection of *S. mansoni* infected mice with helminth parasites showed varying outcomes. *E. caproni* had no protective effect on *S. mansoni* induced liver pathology (42). However, pre-infection with *H. polygyrus* alleviated the schistosome egg-induced hepatic immunopathology (43).

Trichinosis or trichinellosis is a zoonotic parasitic disease of humans and more than 150 animal species transmitted through the consumption of raw or undercooked pork (28, 44, 45). *T. spiralis* is unique among helminths in that adultworms and larvae live in two different habitats within the same host, namely the small intestine and skeletal muscle, respectively (46–48). Therefore, *T. spiralis* is best considered as an intestinal and tissue parasite. In the intestinal phase, the initial T cell response is Th1 that quickly switches to a strong Th2 response, which is also effective against the skeletal muscle infection (49). This parasite has evolved to suppress the host immune response against itself in order to survive (50), but it also suppresses immune responses to autoantigens and allergens (51, 52) and prevents or attenuates malignant cell development and expansion (53). Many aspects of the inhibitory effect of *T. spiralis* on cancer have been investigated both in animals and *in vitro*. Authors reported the antitumor effects of *T. spiralis* lung cancer, colorectal carcinoma, glioma, esophageal carcinoma and mouse ascitic hepatoma (44, 54, 55). *T*. *spiralis* had shown a good immunomodulatory effects in autoimmune diseases using either crude muscle larval antigens, excretory products, or infection (13, 56–60). In the case of allergic diseases, the use of ESPs from *T. spiralis* has also shown promising results in animal models of allergic asthma, a chronic inflammatory respiratory disorder (61, 62).

Although it should be borne in mind that *T. spiralis* infection could be followed by adverse effects like downregulation of T cell responses to viral infection, causing its exacerbation (63), it is important to emphasize that Th2 type of immune response induced by helminths may also mitigate tissue damage by reducing harmful inflammation and enhancing tissue repair (64). We believe that understanding the impact of helminth infections on the development of schistosomiasis and the progression of fibrosis could help identify novel therapeutic approaches for schistosomiasis. To the best of our knowledge, there are no studies on the coinfections of *S. mansoni* and *Trichinella spiralis* [*T. spiralis*]. In the present study, we aimed to assess whether the immune response associating *T. spiralis* infection is protective against *S. mansoni* induced liver pathology or not. In our study, using a mouse model, we show that pre-infection with *T. spiralis* mitigated the formation of *S. mansoni* egg granulomas—in comparison to mice infected with *S. mansoni* only- with subsequent alleviation of liver fibrosis.

## 2 Materials and methods

### 2.1 Animal experiment

Thirty adult male Balb-c mice, each weighing 18–20 g, were obtained from the Schistosome Biological Supply Program at Theodor Bilharz Research Institute, Imbaba, Giza, Egypt. For *S. mansoni* infection, 20 shedding adult B. alexandrina snails (4–6 mm in diameter) were obtained from the Schistosome Biological Supply Centre, Theodor Bilharz Research Institute, Cairo, Egypt. Snails were allowed to shed under light and the fresh exiting cercariae were used to infect the mice. Briefly, the infected snails were kept in a test tube containing distilled water and then exposed to artificial light at 28°C ± 1 for 2 h to induce shedding of cercariae. The number of cercariae was determined by using a dissecting microscope. Generally; three counts were made and the average was used to calculate the number of cercariae per 0.1 ml of the cercarial suspension. For *T. spiralis* infection, *T. spiralis*-infected BALB/c mice were acquired from the Assiut University's Faculty of Medicine in Assiut, Egypt. As previously mentioned, larvae were extracted from the affected muscles (65). To summarize, the infected muscles were subjected to a 12-h mechanical stirrer immersion in a 1,000 ml saline solution containing 20 mL of HCl and 20 g of pepsin at 37°C. The suspension was centrifuged for 2 min at 1,000 rpm in order to liberate the larvae. The material was centrifuged again after being washed with saline (0.9% NaCl). Hemocytometers were used to count the larvae in order to calculate the size of the inoculum needed to infect mice. For use in the animal trials, the sediment containing the larvae was re-suspended in saline containing 1.5% gelatin.

The mice were divided into three groups, each of 10 mice. Group I mice were kept uninfected, whereas group 2 mice was infected with approximately 60 ± 10 *S. mansoni* cercariae by the paddling method, where mice were immobilized without anesthetics and the tail was exposed and immersed in water containing *S. mansoni* cercaria for 45 min (66). Mice in group 3 were orally infected with *T. spiralis* larvae (300 larvae/mouse) then infected with approximately 60 ± 10 *S. mansoni* cercariae by the paddling method at 28^th^ day post *Trichinella* infection (67). All mice were anesthetized with isoflurane by the inhalation route and euthanized by cervical dislocation at the end of 8^th^ week post infection with *S. mansoni*.

### 2.2 Parasitological studies

#### 2.2.1 Determination of *S. mansoni* worm burden

Sacrificed mice were subjected to hepato-portomesenteric perfusion technique to collect adult *S. mansoni* worms, detect sex [male/female/copula], determine worm burden, and then calculate the percentage of reduction of total worms, as described previously (68). Briefly, adult worms from each mouse were recovered in a Petri dish. Males and females were differentiated using a dissecting microscope on basis of the size and color of the parasites in addition to the presence of gynaecophoric canal and tubercles, which are characteristics absent in the female. Male adult worms are clear and female adult worms are longer and darker. Then, males and females recovered from each mouse and each group were counted for calculation of the mean number for male/female/copula in each group.

#### 2.2.2 Determination of egg count in tissues

Small pieces of hepatic and intestinal tissue were weighted, digested overnight in 5 ml of 5% KOH solution, and three samples (each 50 μl) of the digested tissue were examined microscopically to determine the mean of *S. mansoni* egg count (69). Number of eggs/gram tissue, and the percentage reduction in total eggs/gram tissue were calculated according to Kloetzel (70).

### 2.3 Biochemical measurements

Blood was obtained by cardiac puncture and was centrifuged at 600 xg for 10 min to obtain the serum. The levels of alanine aminotransferase (ALT) and aspartate aminotransferase (AST) enzymes in mice sera were measured by Hitachi 7080 Chemistry Analyzer (Hitachi Ltd., Tokyo, Japan) using commercial kits from Randox Laboratories Ltd. (Crumlin, Northern Ireland).

### 2.4 Histopathological studies

#### 2.4.1 Hematoxylin and eosin staining and Masson's trichrome staining

Liver tissue specimens were obtained from all groups of mice and immediately fixed in 10% buffered formalin for 24 h and then dehydrated in increasing concentrations of ethanol and processed for paraffin sectioning. Sections of 4 μm thick were deparaffinized, rehydrated in decreasing concentrations of alcohol, and stained with hematoxylin and eosin (H&E). The H&E staining protocol starting with staining with Harris hematoxylin solution followed by counterstaining with Alcoholic-Eosin solution was strictly followed. Slides were then dehydrated, cleared in Xylene and then mounted and cover slipped. H&E-stained sections were examined for granuloma formation and the associated histopathological changes. The number and sizes of the granulomas in different groups were determined. Mean granuloma number was determined in 10 successive fields of five slides from each mouse, and was accordingly determined in each group (71). Similarly, mean granuloma size in each mouse was calculated by measuring their diameters under the light microscope, equipped with an ocular micrometer. Only granuloma surrounding eggs were measured. The mean diameter was calculated from 10 granuloma, and the mean granuloma size was calculated for each group (71).

For Masson's trichrome (MT), outline steps, such as fixation, grossing, processing, embedding, and sectioning, were performed before MT staining. After deparaffinization and rehydration, the sections were re-fixed in Bouin's solution for 1 h at 56°C to enhance the staining quality. The MT stain procedure includes staining with Weigert's iron hematoxylin, Biebrich scarlet-acid fuchsin, and aniline blue solutions, followed by dehydration in alcohol grades. Then slides were mounted and cover slipped and examined for histopathological evaluation and image analysis.

Using MT staining, paraffin-embedded liver tissue sections were used according to Kiernan (72), to show the density of fibrosis in granulomas. The mean fibrosis area percent in 10 microscopic fields of each specimen was calculated and then the mean percent fibrosis/group was determined and compared between groups.

#### 2.4.2 Immunohistochemical staining

Sections at 4 μm thickness were taken from the previously prepared paraffin-embedded tissue blocks and mounted on glass slides. Sections were then deparaffinized, rehydrated with decreasing concentrations of alcohol, then rinsed with distilled water. Endogenous peroxidase activity was blocked using 0.6% hydrogen peroxide for 10 min. For epitope retrieval, sections were microwaved in citrate buffer (pH 6) for 12 min. Sections were then incubated with anti-α-smooth muscle actin () antibody (α-SMA, ABclonal, catalog no A7248, dilution: 1: 50), interleukin 1 beta (IL 1β, ABclonal, catalog no A16640, dilution: 1: 50), interleukin 6 (IL-6, ABclonal, catalog no A0286, dilution: 1: 50), interleukin 23 (IL-23, ABclonal, catalog no A1613, dilution: 1: 50), tumor necrosis factor-α (TNF-α, ABclonal, catalog no A11534, dilution: 1: 50), transforming growth factor- β (TGF-β, ABclonal, catalog no A16640, dilution: 1: 50), and interleukin 17 (IL-17, ABclonal, catalog no A12454, dilution: 1: 50) for 1 h at room temperature. Sections were washed with TBS containing 0.05% Tween-20 (TBS-T) and were then incubated with HRP-conjugated goat anti-rabbit secondary antibodies (Vivantis Technologies, Malaysia) at a dilution of 1:5,000 for 1 h at 4°C. After washing in TBS-T, the color was developed by incubating sections with 0.05% diaminobenzidine (DAB) and 0.01% H_2_O_2_ for 3 min. Counterstaining was performed with hematoxylin for 30 seconds, and sections were then examined by light microscopy. Negative controls were obtained by omitting the primary antibody.

Hepatocytes with cytoplasmic reaction to the antibodies were considered positive. Semi-quantitative analysis of positively stained tissue sections was performed through modified Allred scoring system guidelines. The percentage of positive cells was estimated in 3 different fields (200x) and the mean percentage (±SD)/group was calculated. Individual scores of the percentage of positive cells (0–5) and the intensity of cytoplasmic staining (0–3) were summed up to obtain the final scores. The scoring of percentage of positive cells was set as follows: 1: < 10% positive cells; 2: 10%−20% positive cells; 3: 20%−50% positive cells; 4: 50%−70% positive cells; and 5: more than 70% positive cells. The scoring of staining intensity was determined as follows: 1: weak; 2: moderate; and 3: strong.

### 2.5 Statistical analysis

The Statistical Package for Social Sciences (SPSS) version 20 for Windows was used to analyze the acquired data. All parameters were shown as mean ± standard deviation (SD). ANOVA (analysis of variance) and Independent sample *T* test were used for statistical comparison between the groups. A *p*-value of < 0.05 was regarded as statistically noteworthy.

## 3 Results

### 3.1 Pre- infection with *T. spiralis* reduced adult *S. mansoni* worm count as well as intestinal and hepatic egg burden

Worm burden of *S. mansoni* was determined at the end of the 8^th^ week post *S. mansoni* infection. Mice pre-infected with *T. spiralis* showed statistically significant reduction of the worm count in comparison to mice infected with only *S. mansoni* [R (percent of reduction) = 82.4%, *P* = 0.045 and *P* = 0.002 for males and females adults worms respectively; Supplementary Table 1].

In the same context, there was a statistically significant reduction in the count of eggs per gram of intestine and liver in mice pre-infected with *T. spiralis* compared to those infected with *S. mansoni* alone (*P* = 0.009*, P* = 0.014*, P* = 0.009 for intestinal, liver and total egg count respectively; Supplementary Table 2).

### 3.2 Pre- infection with *T. spiralis* reduced ALT and AST levels in the sera of *S. mansoni*-infected mice

**Figure 1:**
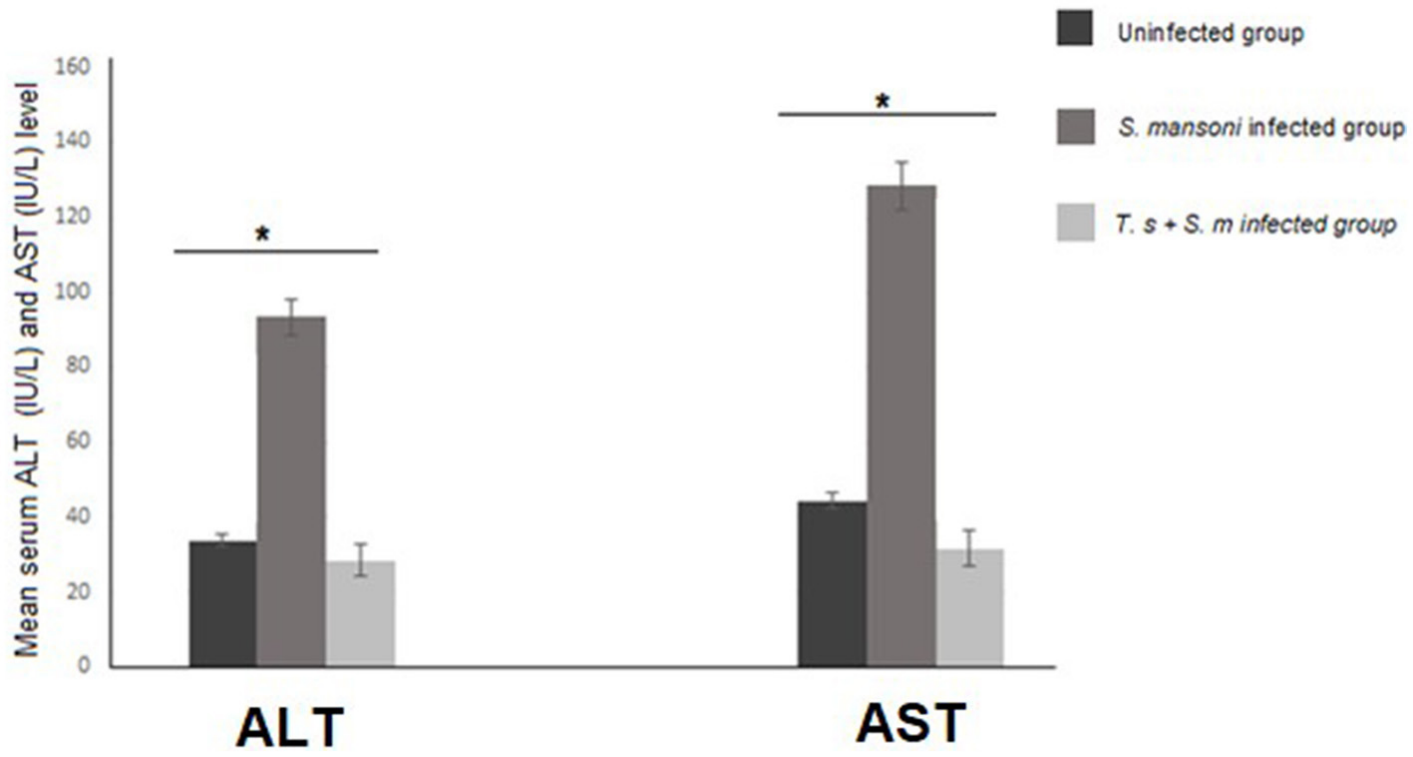
ALT and AST serum levels in mice infected with *S. mansoni* compared to uninfected mice and mice that were pre-infected with *T. spiralis*. Values represent mean percentage ± SD, and data were analyzed using ANOVA with Tukey corrections as a *post hoc* test. Asterisks [*] indicate significant difference; *P* = 0.007 for ALT and *P* = 0.004 for AST.

Next, we measured the levels of serum ALT and AST as markers for liver function. We found that serum ALT and AST levels were higher in the *S. mansoni*-infected mice than in mice that were pre-infected with *T. spiralis* (with statistical significance *P* = 0.007 and 0.004 for AST and respectively, Figure 1).

### 3.3 Pre-infection with *T. spiralis* reduced the number and diameter of granulomas in *S. mansoni*-infected mice

**Figure 2:**
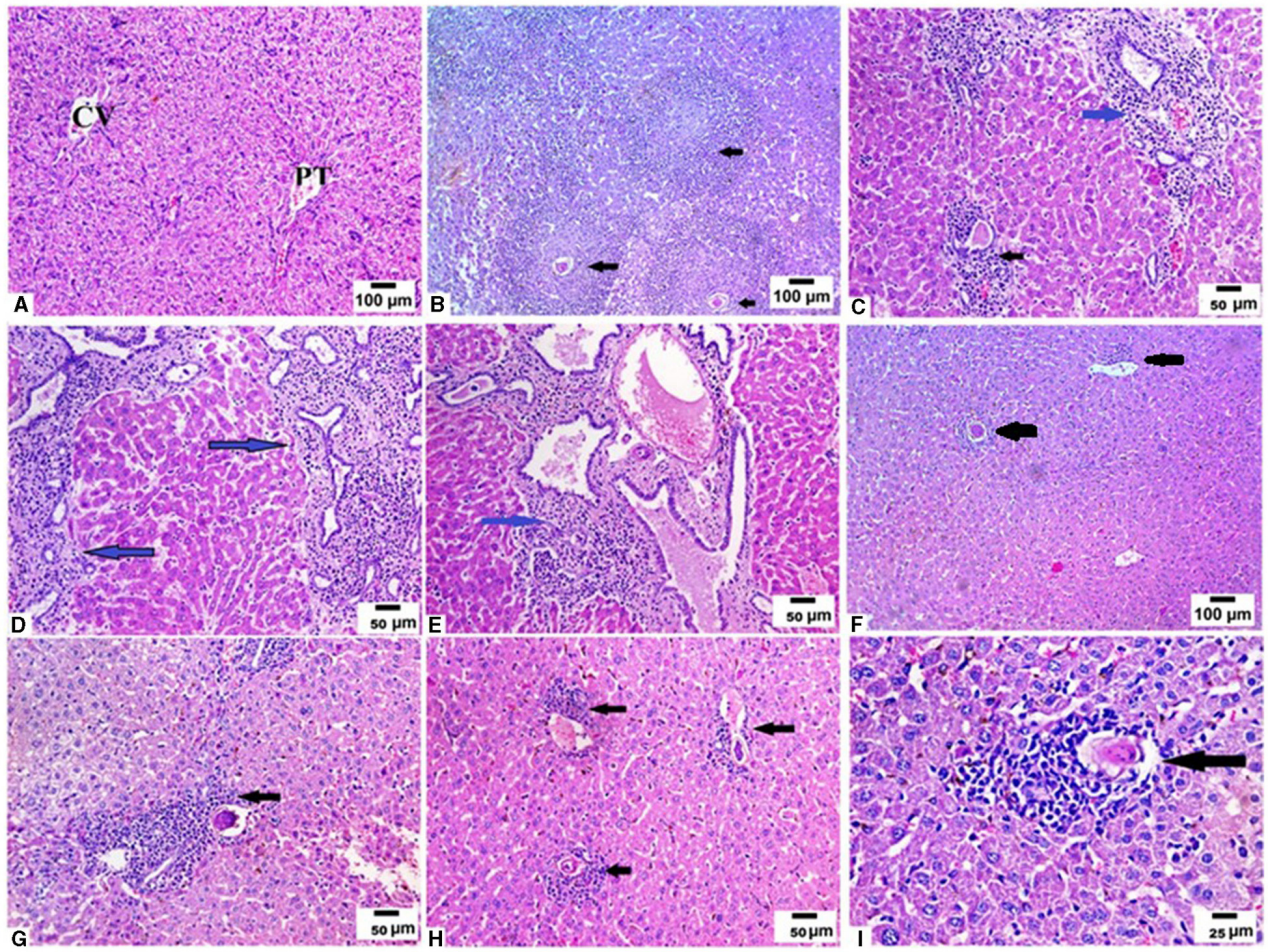
Pre-infection with *T. spiralis* reduced the size and number of granulomas in *S. mansoni* infected mice. Liver sections of different animal groups were stained with H&E stain. **(A)** Representative photomicrograph of liver sections of uninfected mice showing preserved hepatic lobular architecture with small portal tracts (PT), central veins (CV), and intact hepatocytes (200x). **(B)** Representative photomicrograph of liver sections of mice infected only with *S. mansoni* showing severe granulomatous inflammation around *S. mansoni* egg (black arrow) (100x). **(C–E)** Representative photomicrographs of liver sections of mice infected only with *S. mansoni* showing thickened portal tracts with mononuclear cellular infiltration (blue arrows) severe granulomatous inflammation around *S. mansoni* egg (black arrow) (200x). **(F)** Representative photomicrographs of liver sections of mice pre-infected with *T. spiralis* followed by *S. mansoni* showing markedly reduced granuloma size (black arrows) (100x). **(G, H)** Representative photomicrographs of liver sections of mice pre-infected with *T. spiralis* followed by *S. mansoni* showing cellular egg granulomas (black arrows) with intact central ova with markedly reduced granuloma size (200x). **(I)** Representative photomicrograph of liver sections of mice pre-infected with *T. spiralis* followed by *S. mansoni* showing mild granulomatous inflammation around *S. mansoni* egg (black arrow) (400x).

**Figure 3:**
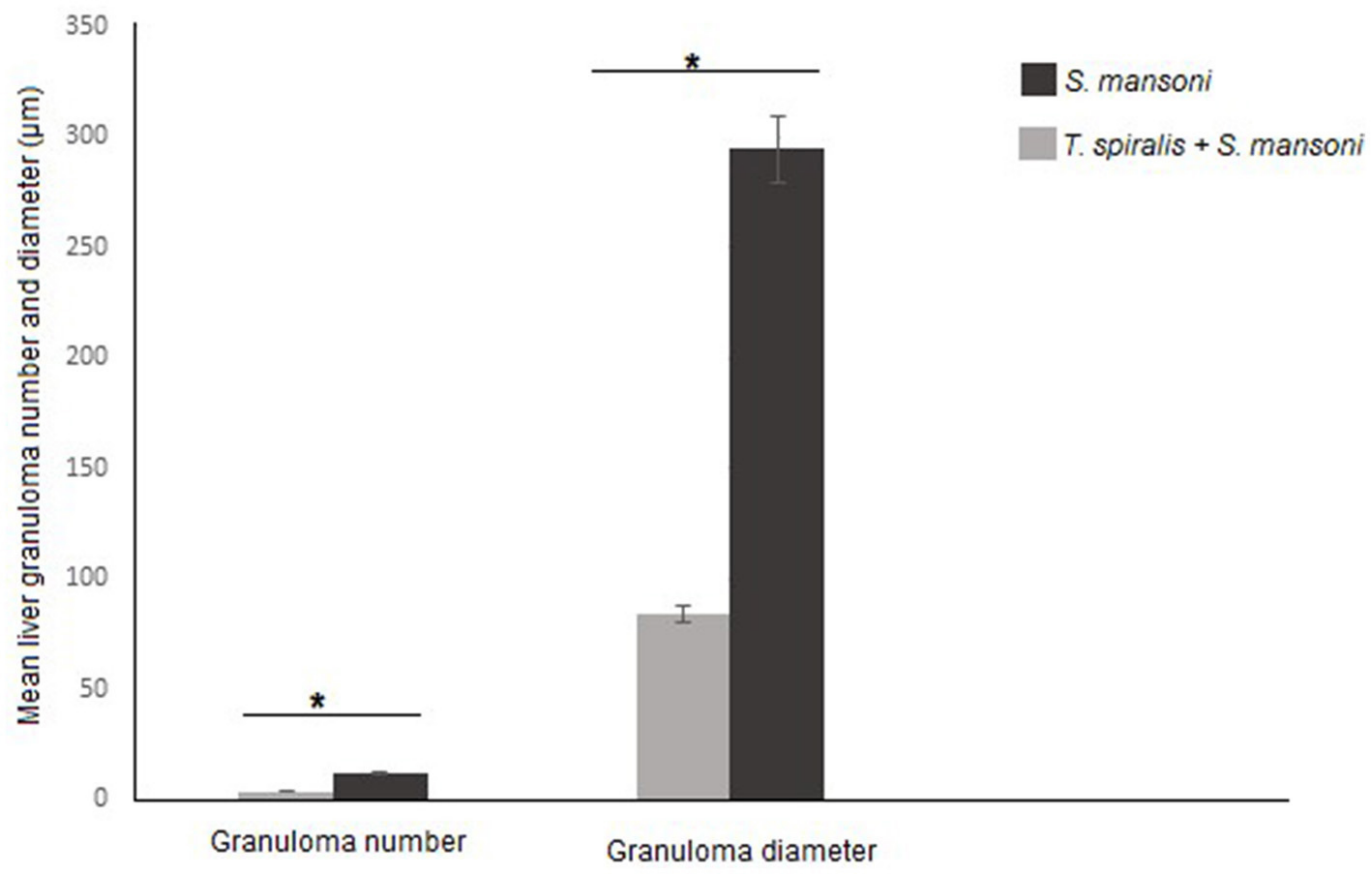
Granulomas number and size in livers of mice that were pre-infected with *T. spiralis* followed by *S. mansoni* infection compared to *S. mansoni* monoinfection. Asterisks (*) indicate a statistically significant reduction in the number (*P* = 0.014) and diameter of granulomas (*P* = 0.014).

We examined the livers extracted from all animal groups using H&E staining. Liver sections of uninfected mice showed normal hepatocytic architecture with no inflammatory cells in between or surrounding the central vein, normal hepatic lobules, and bile ducts (Figure 2A). Infection with *S. mansoni* caused marked granulomatous inflammation (Figure 2B; black arrows). Hepatic granulomas were of two types: fibrocellular (75%) and cellular granulomas (25%). Cellular granulomas were made up of bilharzial ova and adult worms surrounded by lymphocytes, eosinophils, histiocytes, macrophages and plasma cells with altered liver architecture (Figures 2C–E; blue arrows). On the contrary, mice infected with *T. spiralis* prior to *S. mansoni* infection showed alleviation of *S. mansoni-*induced pathological alterations with marked reduction of granuloma size (Figure 2F; black arrows). All granulomas recorded in this group were of the cellular type. Granulomas consisted of lymphocytes, eosinophils, histiocytes, macrophages, and plasma cells surrounding bilharzial ova and adult worms with disrupted liver architecture (Figures 2G–I; black arrows). Pre-infection with *T. spiralis* significantly reduced the number of *S. mansoni* induced hepatic granulomas (mean ± SD = 11.8.3 ± 1.22 in *S. mansoni* only-infected mice vs. 3.4 ± 1.1 in mice priorly infected with *T. spiralis*) (Figure 3). Hepatic granuloma diameters were significantly reduced in mice that were pre-infected with *T. spiralis* compared to *S. mansoni* only-infected mice (mean ± SD = 294.3 ± 16.22) in *S. mansoni* only-infected mice vs. (mean ± SD = 84 ± 11) in mice pre-infected with *T. spiralis* (Figure 3).

### 3.4 Pre-infection with *T. spiralis* alleviated *S. mansoni* induced liver fibrosis

**Figure 4:**
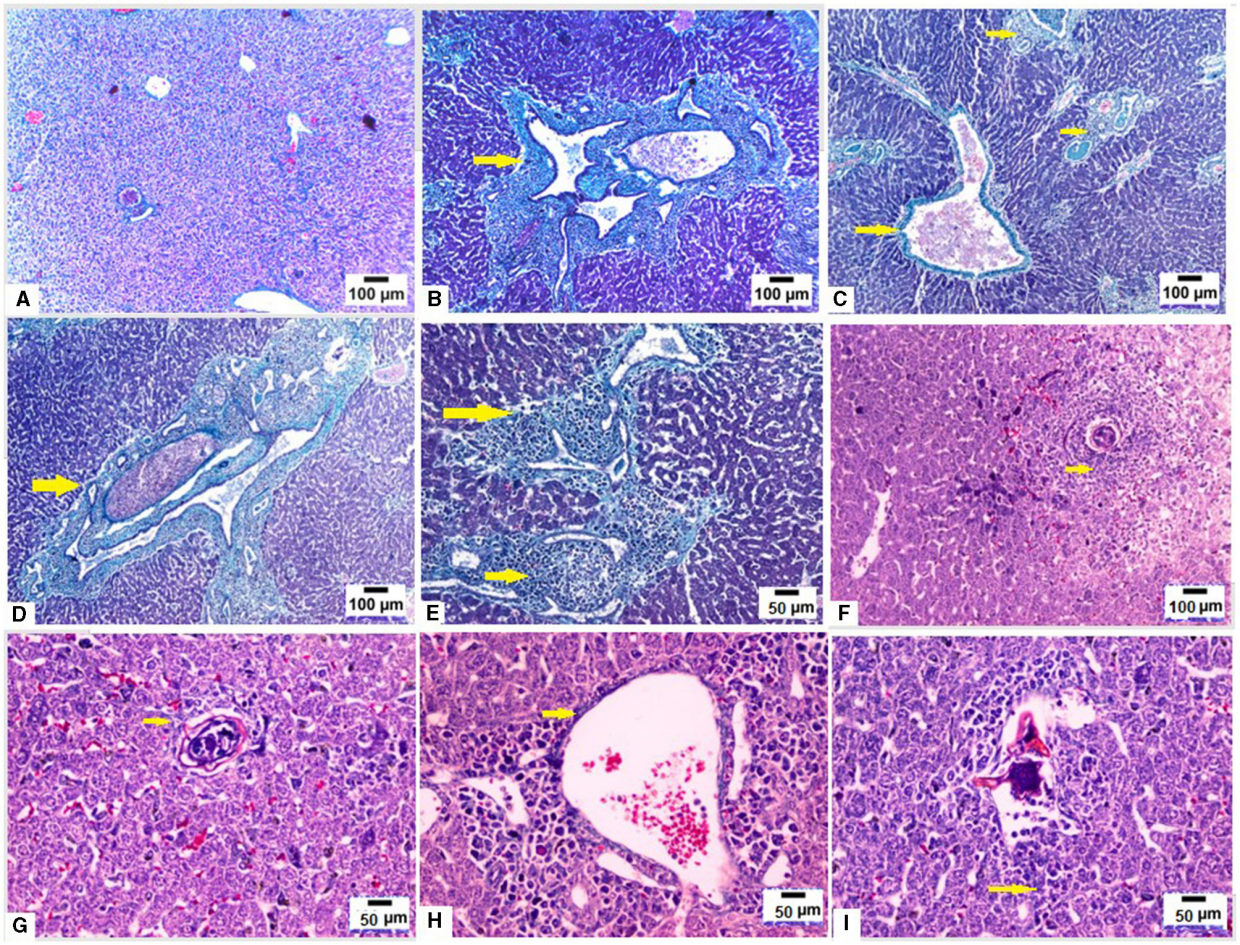
Pre-infection with *T. spiralis* reduced hepatic fibrosis due to *S. mansoni* infected mice. Liver sections of different animal groups were stained with Masson's trichrome stain. **(A)** Representative photomicrograph of liver sections of uninfected mice showing no fibrosis (100x). **(B–D)** Representative photomicrographs of liver sections of mice infected only with *S. mansoni* showing marked expansion of portal tracts by fibrous tissue and mononuclear inflammatory cells (yellow arrows) (100x). **(E)** Representative photomicrograph of liver sections of mice infected only with *S. mansoni* showing extensive deposition of fibrous tissue (200x). **(F)** Representative photomicrograph of liver sections of mice pre-infected with *T. spiralis* followed by *S. mansoni* showing cellular granuloma with no evidence of fibrosis (100x). **(G)** Representative photomicrograph of liver sections of mice pre-infected with *T. spiralis* followed by *S. mansoni* showing cellular granuloma with minimal fibrosis (yellow arrows) (200X). **(H, I)** Representative photomicrographs of liver sections of mice pre-infected with *T. spiralis* followed by *S. mansoni* showing minimal delicate fibrosis (yellow arrows) (200x).

We used MT stain to evaluate fibrosis in hepatic tissues of mice from different groups. Liver sections from uninfected mice showed normal liver architecture and no fibrosis (Figure 4A). On the other hand, infection with *S. mansoni* resulted in large fibrocellular granulomas with central eggs, marked fibrosis and a significant amount of collagen deposition in concentric manner within those granulomas in addition to extensive fibrous collagen deposition between the portal vein and lobules (Figures 4B–E; yellow arrows). On the contrary, pre- infection with *T. spiralis* treatment significantly decreased collagen fiber accumulation, suggesting that *T. spiralis* infection prevented hepatic fibrosis (Figures 4F–I).

**Figure 5:**
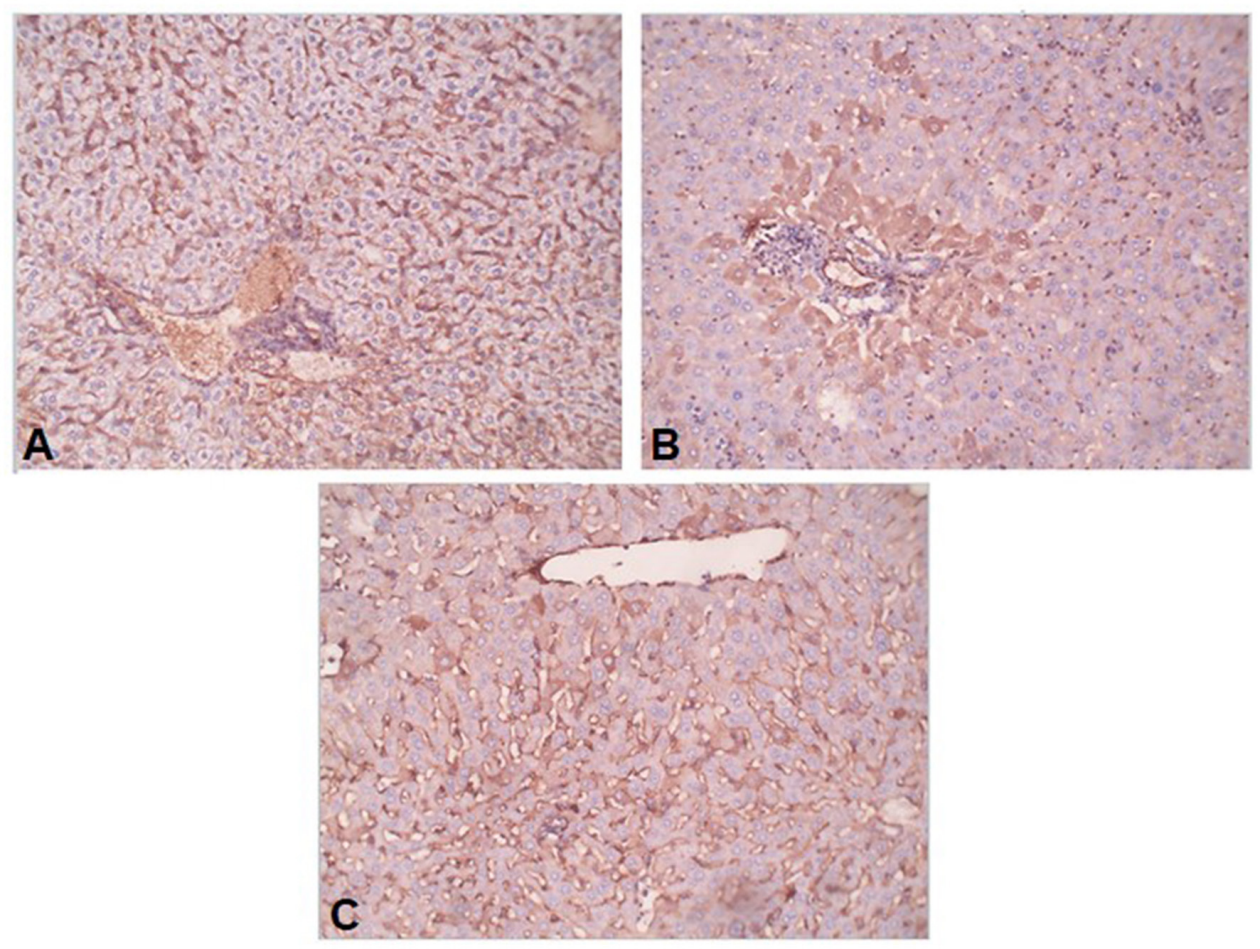
Preinfection with *T. spiralis* alleviated the liver fibrosis in *S. mansoni*-infected mice. Representative photomicrographs of liver sections stained for α-SMA: **(A)** Uninfected mice liver section showing weak reaction in sinusoids only, **(B)** *S. mansoni-*infected mice liver section showing focal strong expression of α-SMA in peri-portal hepatocytes, and **(C)** Liver section of mice pre-infected with *T. spiralis* followed by *S. mansoni* infection showing no expression of α-SMA in hepatocytes. Magnification is 200x.

Conversion of hepatic stellate cells (HSCs) into fibroblasts is the key event in the process of liver fibrosis. The expression of α-SMA is commonly used as a hallmark of activated HSCs. In our study, the uninfected mice livers showed no expression of α-SMA or low expression that was limited to the walls of the central vein (Figure 5A). In the *S. mansoni*-infected mice, intense α-SMA immunostaining was observed in the central and portal tract areas (Figure 5B). Interestingly, pre-infection with *T. spiralis* reduced α-SMA expression compared to *S. mansoni*-infected mice (Figure 5C), indicating inhibition of HSC activation.

### 3.5 Pre-infection with *T. spiralis* reduced inflammation in *S. mansoni*-infected mice

**Figure 6:**
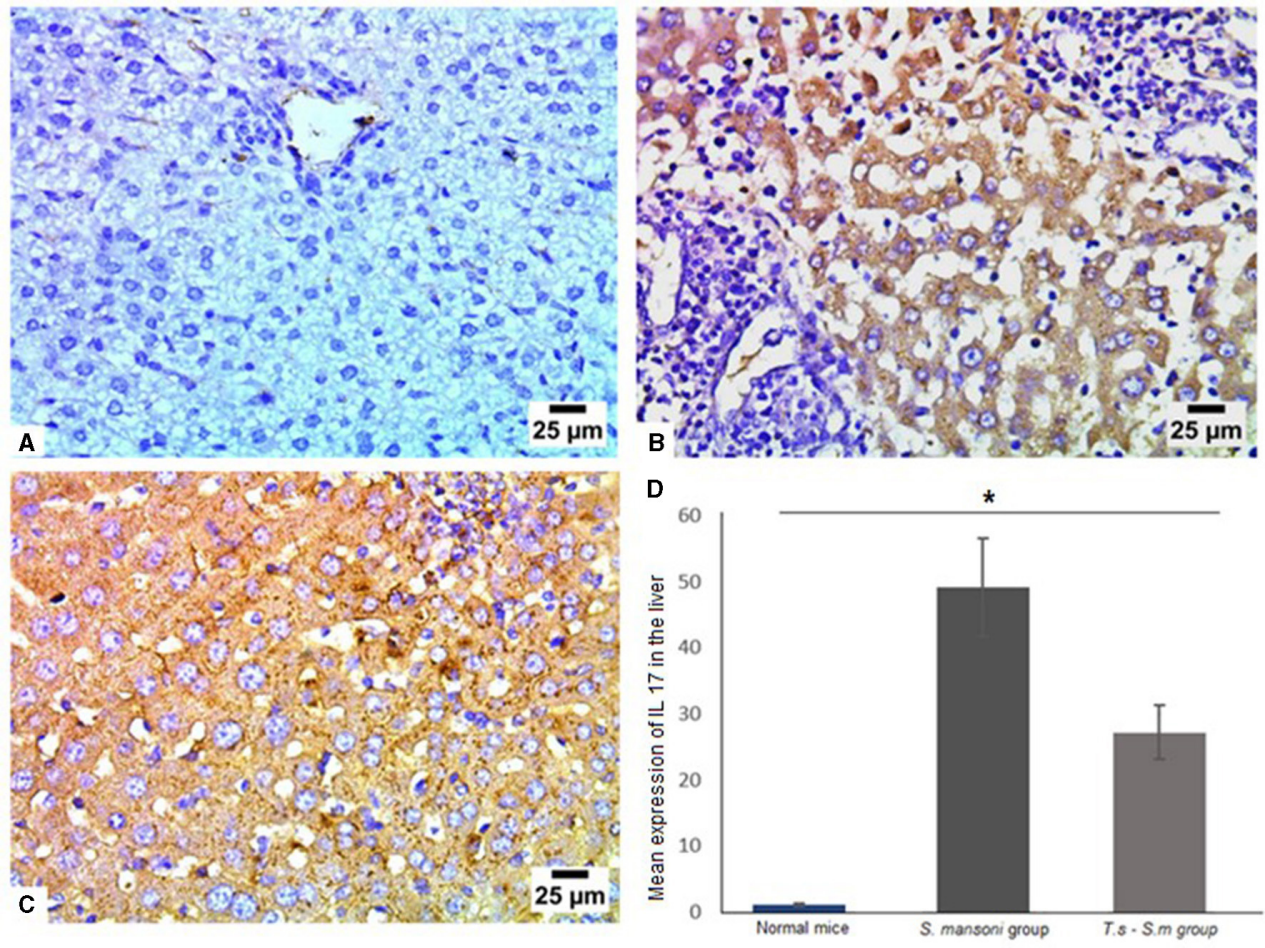
Pre-infection with *T. spiralis* reduced IL-17 expression in *S. mansoni*-infected mice. Representative photomicrographs of liver sections stained for IL-17: **(A)** Uninfected mice liver tissue showing negative expression of IL-17, **(B)** liver tissue of *S. mansoni*-infected mice showing intense expression of IL-17 and **(C)** Liver tissue of *T. spiralis*-pre-infected mice showing significant reduction of IL-17 expression. Magnification is 400x. **(D)** Quantification of IL-17 expression in different mice groups. Values represent mean percentage ± SD, and data were analyzed using ANOVA with Tukey corrections as a *post hoc* test. Asterisk (*) indicates a statistically significant difference; *P* = 0.012.

Mechanisms underlying the pathology in schistosomiasis are not well-defined. Animal studies identified a moderate type 1 helper [Th1] response to parasite antigens, but a robust Th2 response to egg-derived antigens dominates and promotes fibrogenesis in the liver (73, 74). Th17 cells produce several cytokines, including IL-17, and have demonstrated profibrogenic roles in different experimental models of hepatic, pulmonary, and myocardial fibrosis (75–77). Based on the above findings, we examined the expression of various cytokines in liver tissues of all mice groups (Figures 6A–D). Mice infected with *S. mansoni* only showed high expression of IL-17 (mean percentage = 49 ± 15, Figures 6B and D). There was a marked reduction in IL-17 production in mice that were pre-infected with *T. spiralis* (Figures 6C and D, *P* = 0.012, mean percentage ± SD = 29 ± 13.5).

**Figure 7:**
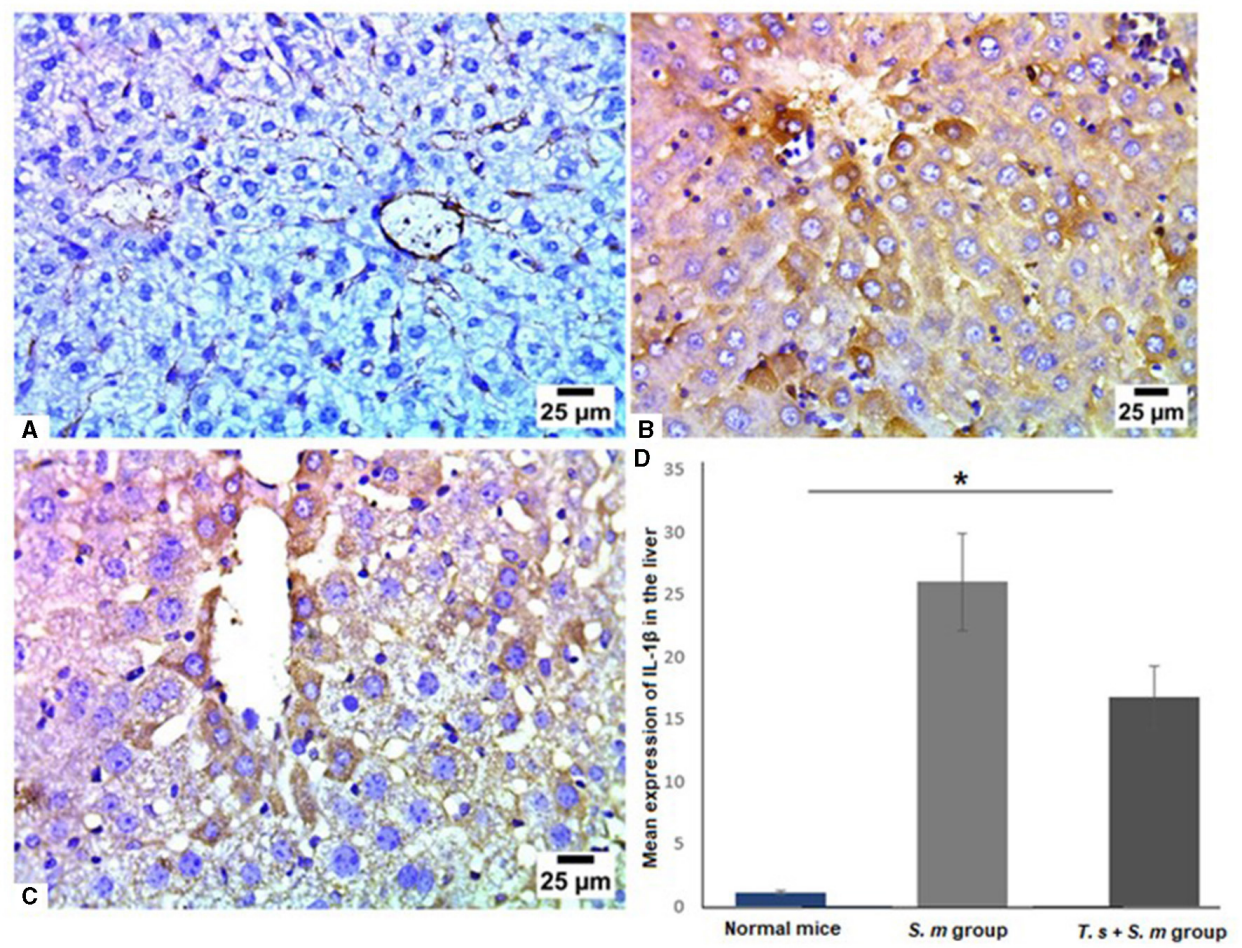
IL-1β was significantly reduced in liver tissues of *T. spiralis* pre-infected mice. Representative photomicrographs of liver sections stained for IL-1β: **(A)** Uninfected mice liver tissue showing negative expression of IL-1β, **(B)** *S. mansoni*-infected mice liver tissue showing intense expression of IL-1β and **(C)** Liver tissue of *T. spiralis*- pre-infected mice showing significant reduction of IL-1β expression. Magnification is 400x. **(D)** Quantification of IL-1β expression in different mice groups. Values represent mean percentage ± SD, and data were analyzed using ANOVA with Tukey corrections as a *post hoc* test. Asterisk (*) indicates a statistically significant difference; *P* = 0.005.

IL-1β is a significant mediator of tissue damage and plays an essential role in the progression of schistosomiasis (78–81). Therefore, we examined IL-1β levels in livers of mice of different groups (Figures 7A–D). Similar to IL-17, we found that IL-1β was highly expressed in *S. mansoni* only infected mice (Figures 7B and D, mean percentage ± SD = 27.5 ± 11.5). IL-1β was significantly downregulated in the livers of mice that were pre-infected with *T. spiralis* compared to *S. mansoni*-infected mice (Figures 7C and D, mean percentage ± SD = 15± 6.7, *P* = 0.005).

**Figure 8:**
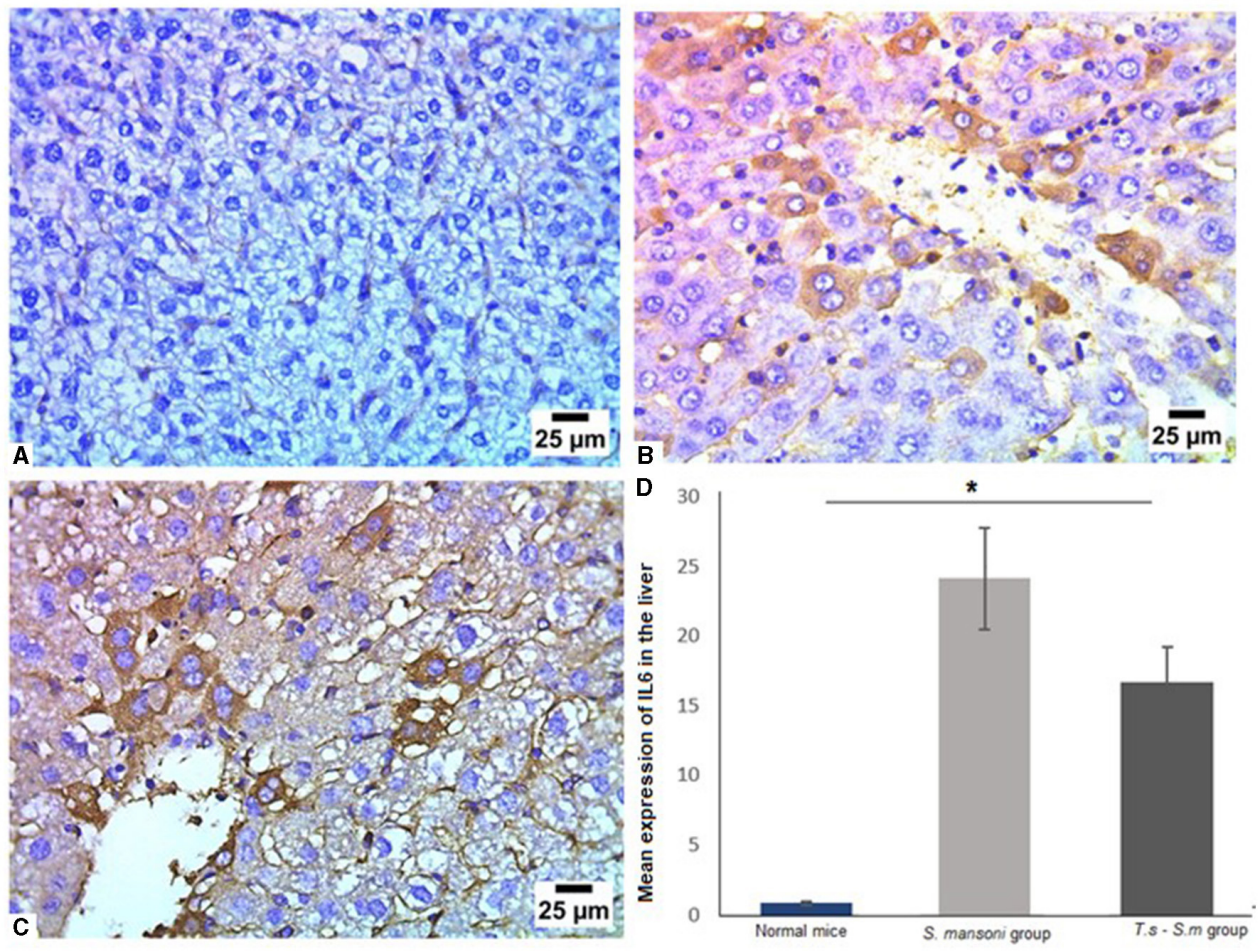
Pre-infection with *T. spiralis* significantly reduced IL-6 in liver tissues of *S. mansoni*-infected mice. Representative photomicrographs of liver sections stained for IL-6: **(A)** Uninfected mice liver tissue showing negative expression of IL-6, **(B)** Liver tissue of *S. mansoni*-infected mice showing positive expression of IL-6 and **(C)** Liver tissue of *T. spiralis*-pre-infected mice showing significant reduction of IL-6 expression. Magnification is 400x. **(D)** Quantification of IL-6 expression in different mice groups. Values represent mean percentage ± SD, and data were analyzed using ANOVA with Tukey corrections as a *post hoc* test. Asterisk (*) indicates a statistically significant difference; *P* = 0.04.

Furthermore, we examined the IL-6 levels in liver tissues of different mice groups since it has been reported as a major fibrogenic agent by regulating neutrophil transport, chemokine production, and leukocyte apoptosis (82–85). Our data show that liver tissues of mice that infected only with *S. mansoni* showed high expression of IL-6 (Figures 8A, B and D, mean percentage ± SD = 24.6 ± 10.3). Meanwhile, mice group which were pre-infected with *T. spiralis* had significantly lower levels of IL-6 compared to mice infected with *S. mansoni* alone (Figures 8C and D, mean percentage ± SD = 16.7 ± 3.5, *P* = 0.04).

**Figure 9:**
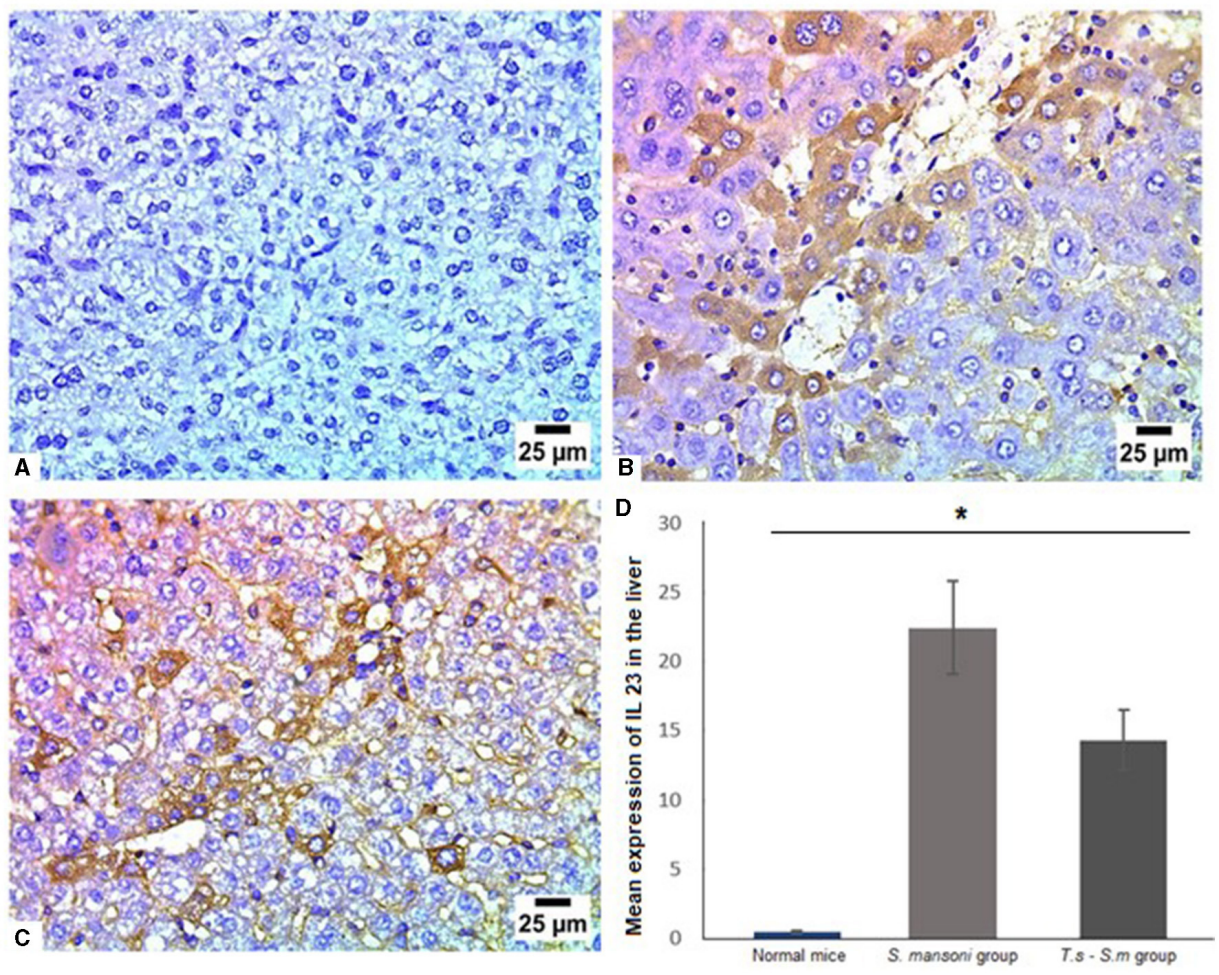
IL-23 had significantly lower levels in liver tissues of *S. mansoni*-infected mice that were pre-infected with T. spiralis. Representative photomicrographs of liver sections stained for IL-23: **(A)** Uninfected mice liver tissue showing no expression of IL-23, **(B)** Liver tissue of *S. mansoni*-infected mice showing high expression of IL-23 and **(C)** Liver tissue of *T. spiralis*-pre-infected mice showing significant reduction of IL-23 expression. Magnification is 400x. **(D)** Quantification of IL-23 expression in different mice groups. Values represent mean percentage ± SD, and data were analyzed using ANOVA with Tukey corrections as a *post hoc* test. Asterisk (*) indicates a statistically significant difference; *P* = 0.032.

Several studies had identified IL-23 as an important pro-inflammatory cytokine involved in inducing Th17 cell differentiation and fibrogenic response (86, 87). Like other cytokines examined in this study, our results showed *S. mansoni* infected mice showed high levels of IL-23 (Figures 9A, B and D, mean percentage ± SD = 22.9 ± 9.7), with a a significant reduction in the expression of IL-23 in *T. spiralis* pre-infected mice compared to mice infected with *S. mansoni* alone (Figures 9C and D, mean percentage ± SD = 14.9 ± 3.2, *P* = 0.032).

**Figure 10:**
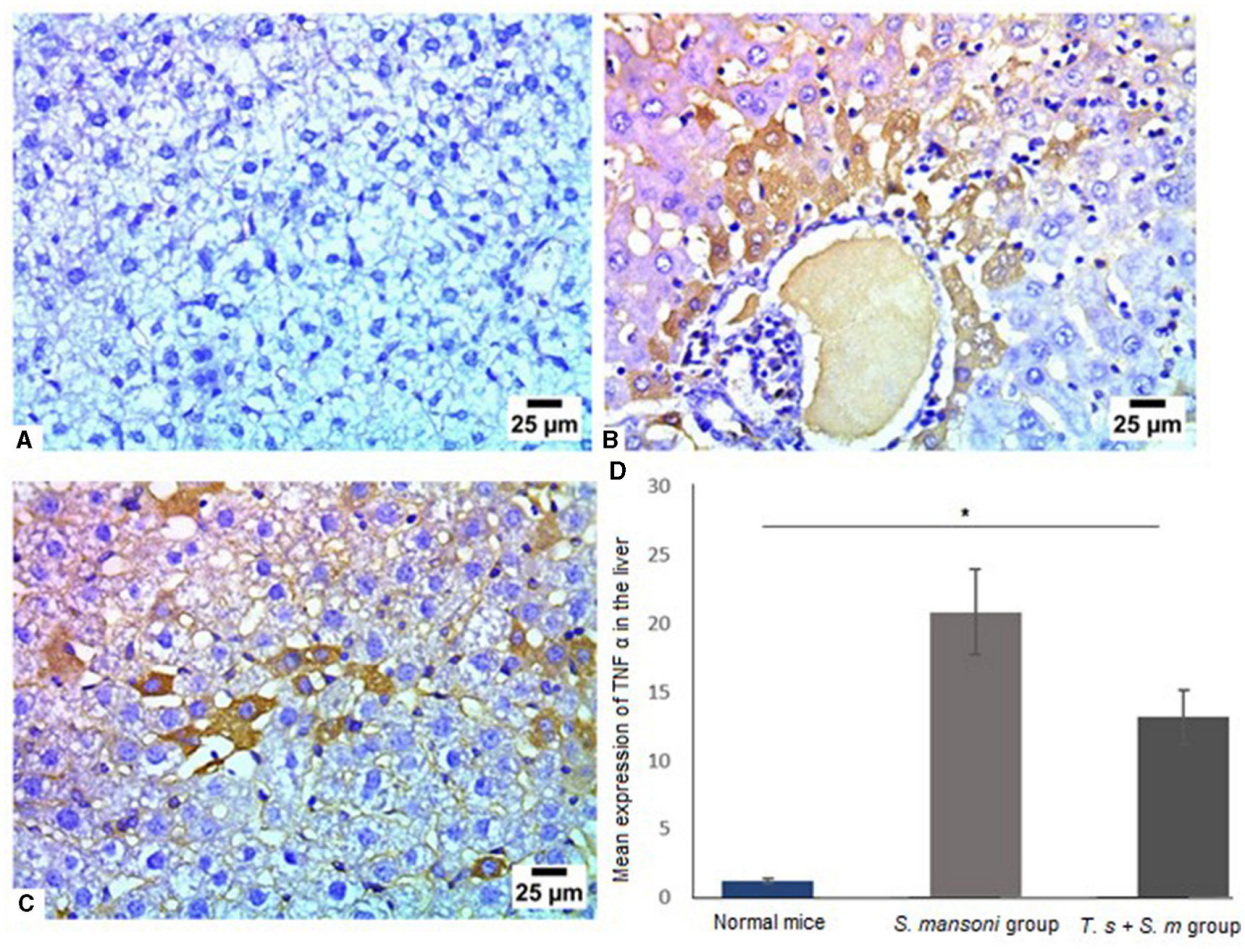
Pre-infection with *T. spiralis* significantly reduced TNF-α in liver tissues of *S. mansoni*-infected mice. Representative photomicrographs of liver sections stained for TNF-α: **(A)** Uninfected mice liver tissue showing no expression of TNF-α, **(B)** Liver tissue of *S. mansoni*-infected mice showing positive expression of TNF-α and **(C)** Liver tissue of *T. spiralis*-pre-infected mice showing mild positive expression of TNF-α. Magnification is 400x. **(D)** Quantification of TNF-α expression in different mice groups. Values represent mean percentage ± SD, and data were analyzed using ANOVA with Tukey corrections as a *post hoc* test. Asterisk (*) indicates a statistically significant difference; *P* = 0.031.

Next, we aimed to examine the expression of TNF-α in liver tissue of different mice groups since it is an important mediator of murine granuloma formation and hepatic fibrosis (88). In line with our findings in this study, TNF-α was markedly high in mice infected with *S. mansoni* (Figures 10A, B and D, mean percentage ± SD = 12 ± 1.5). Significant reduction in TNF-α expression in liver tissues of mice that were pre-infected with *T. spiralis* in comparison to mice infected with *S. mansoni* alone (Figures 10C and D, mean percentage ± SD = 9 ± 3.1, *P* = 0.031).

**Figure 11:**
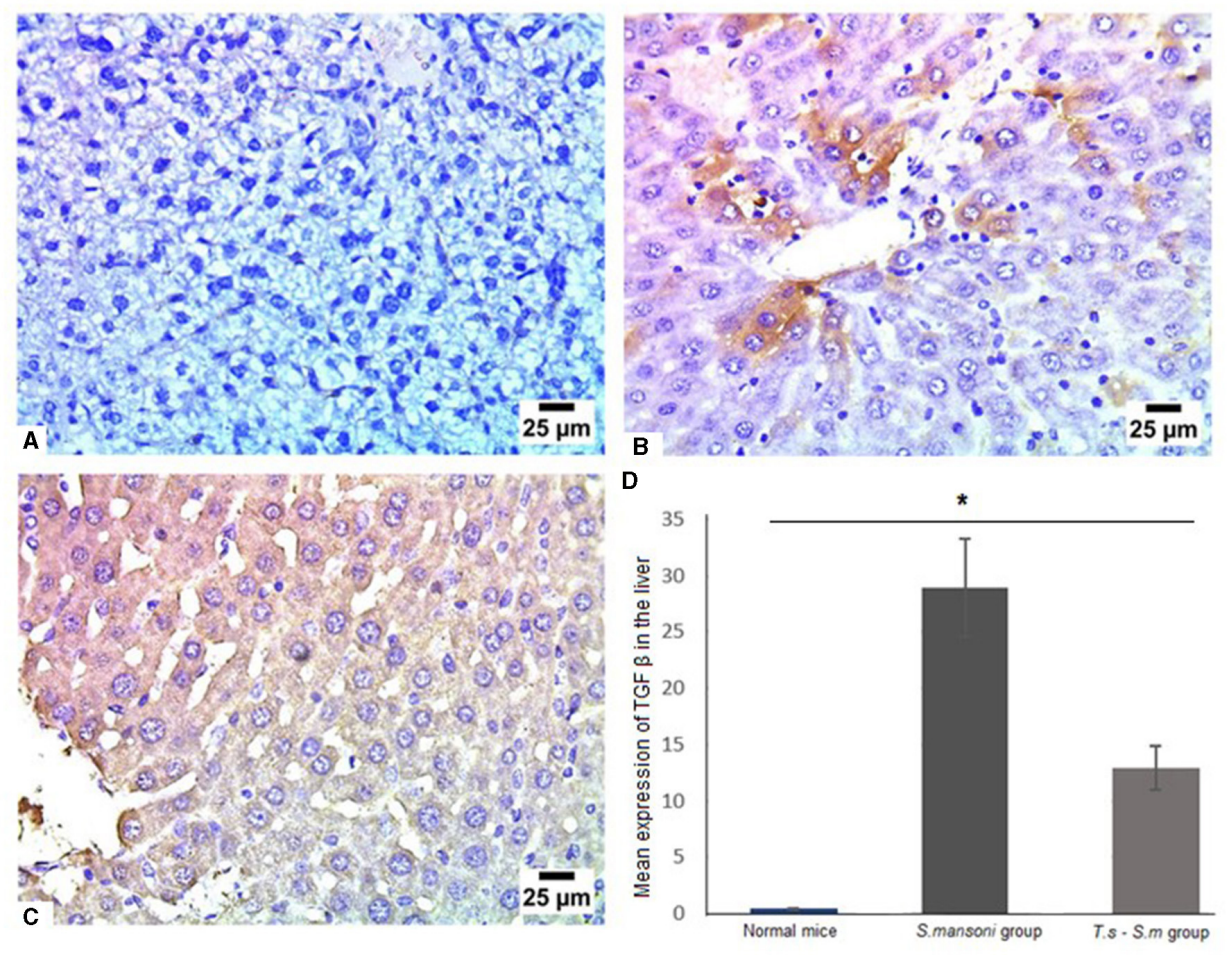
TGF-β was markedly reduced in liver tissues of *T. spiralis*-pre-infected mice. Representative photomicrographs of liver sections stained for TGF-β: **(A)** Uninfected mice liver tissue showing no expression of TGF-β, **(B)** Liver tissue of *S. mansoni*-infected mice showing moderate expression of TGF-β and **(C)** Liver tissue of *T. spiralis*-pre-infected mice showing lower expression of TGF-β. Magnification is 400x. **(D)** Quantification of TGF-β expression in different mice groups. Values represent mean percentage ± SD, and data were analyzed using ANOVA with Tukey corrections as a *post hoc* test. Asterisk (*) indicates a statistically significant difference; *P* = 0.041.

The production of TGF-β may modulate inflammation and regulate fibrogenesis in response to *S. mansoni* eggs. Several investigators indicated that TGF-β is a regulatory cytokine, that is mainly produced by regulatory T cells, which provides an effective mechanism of control of the progression of fibrosis (88, 89). Our data showed that infection of mice with *S. mansoni* increased the level of TGF-β in liver tissues compared to uninfected mice group (Figures 11A, B and D, mean percentage ± SD = 28.7 ± 7.5). However, prior *T. spiralis* infection significantly reduced the production of TGF-β (Figures 11C and D, mean percentage ± SD = 12 ± 2.5, *P* = 0.041). All our findings support the protective role of *T. spiralis* prior infection against the pathological effects of *S. mansoni* infection.

## 4 Discussion

In the case of infection with schistosomes, there is predictable severe disease in about 5% to 10% of the population (43). Granuloma formation and fibrosis are the major causes of morbidity and mortality in association with schistosomiasis. This process may lead to fibrosis with excessive accumulation of collagen and other extracellular matrix proteins in the periportal space (90). The immunopathology in schistosomiasis is mediated by CD4 effector T cells (91).

In this study, we tested the hypothesis that low pathology is at least in part determined by coinfection with intestinal nematodes. Based on the observations that nematode coinfection is prevalent in areas where schistosomiasis is endemic and that nematode infection creates a host immune environment associated with attenuated incidence of CD4 T-cell-dependent autoimmune diseases (5).

Using the murine model of schistosomiasis, we show here that pre-infection with *T. spiralis* parasitic nematode caused a significant reduction in the number of recovered worms, egg count in both the intestine and liver tissues. Regarding *S. mansoni* induced liver pathology, pre-infection with *T*. *spiralis* caused a significant reduction of both number and size of the hepatic granulomatous inflammation caused by schistosome eggs when compared with *S. mansoni* only infected mice. Moreover, *S. mansoni* induced hepatic fibrosis was markedly reduced in mice pre-infected with *T. spiralis* as manifested with the low deposition of collagen in hepatic sections stained with MT in addition to the low expression of α-SMA antibodies.

The production of fibrosis, which are key signs of both chronic and advanced schistosomiasis, depends on different key cytokines (36). In the present study, the decrease in granulomatous reaction and subsequent fibrosis was accompanied by a marked decrease in the levels of IL-17, IL-1B, IL-6, TGF-β, IL 23, and TNF-α. These cytokines are correlated with the immunopathology of schistosomiasis and its drive (92–95). In particular, the proinflammatory function of IL-17, which induces chemokine-mediated leukocyte recruitment, has also been demonstrated in the context of other infectious and autoimmune diseases (10, 11, 96). IL-17 production is associated with a distinct subset of CD4 T cells, Th17 cells (77, 97), which are variously promoted by an array of innate immunocyte-derived cytokines, including IL-6, TGF-β, IL-23, and IL-1β (98–103).

IL-17 was defined as a main player in the process of fibrosis in different experimental models of hepatic, pulmonary, and myocardial fibrosis (75, 76). IL-17-producing cells contribute to the hepatic granulomatous inflammation and subsequent fibrosis in addition to the Th1, Th2, and Th17 associated cytokines (104). In the present study, IL-17 was significantly high in mice infected with *S. mansoni* and reduced in mice pre-infected with *T. spiralis*. Similar results have been obtained by previous research which observed that the level of IL-17 was increased in the injured liver compared to control animals (105–107). The increased level of IL-17 facilitates the influx of inflammatory cells, drives the expression of profibrogenic growth factors and activates hepatic stellate cells in the liver (31, 108). The liver infiltrating inflammatory cells in turn induce the production of profibrotic cytokines such as TNF-α, IL-6, IL-1, and TGF-β1 (109). On the other hand, a large body of articles stated that those cytokines involving IL-6, TGF-β, IL-23, and IL-1β are incorporated in the expression of the Th17-specific transcription factor RORγt (98, 99, 101, 110). So, we measured the level of IL-23, it was markedly high in *S. mansoni* only infected mice with significant reduction in mice pre-infected with *T. spiralis*. Our results are in consistency with other authors who reported the role of IL-23 in the immunopathology of schistosomiasis (92, 110).

Regarding IL-6, our results showed that schistosomiasis resulted in high expression of IL-6 which was significantly reduced in mice pre-infected with *T. spiralis*. In line with this result, previous studies have reported high levels of IL-6 in schistosomiasis infected subjects (111–113). Moreover, it has been postulated that the induction of Th17 cells is triggered through simultaneous stimulation with IL-6 (99, 103).

Like IL-6, TGF-β is also a necessary factor for the early differentiation of Th17 cells, and this cytokine induces the expression of the master transcription factor, Foxp3, that is needed for the differentiation of regulatory T cells. So, we examined TGF-β in mice groups. In the current study, infection of mice with *S. mansoni* caused pronounced elevations in serum TGF-β levels, which was reduced in mice pre-infected with *T. spiralis*. Similar results obtained by several studies have reported high level of TGF-β in Schistosomiasis (114, 115). This cytokine is considered a multifunctional cytokine that regulates biological processes such as inflammation, development, and differentiation of many cell types, tissue repair, and tumor genesis. It is also associated with pro-inflammatory responses and immunosuppressive activities (116) and participates in the process of Th17 cells differentiation (117). In the evolution of the granulomatous response to the *S. mansoni* eggs the production of TGF-β may modulate inflammation and regulate fibrogenesis. Several investigators indicated that TGF-β is a regulatory cytokine that is mainly produced by regulatory T cells which provides an effective mechanism of control of the progression of fibrosis (89).

In the same text, IL-1β was reported to promote clonal expansion in an inflammatory environment (102, 118). In the present study, our results demonstrated significant reduction of IL-1β which may also be attributed to reduction of IL-6 level (118). Our results are in accordance with other studies which demonstrated that IL-1β is an important participant, along with other cytokines, and controls the progression from liver injury to fibrogenesis through activation of HSCs (119, 120).

The result of this study showed decreased level of TNF-α after it has been markedly increased due to *S. mansoni* infection in mice. In experimental models, authors attribute to the TNF-α proinflammatory and profibrogenic effects that may aggravate the disease (90). Our results confirmed previously published data that showed that cases of severe portal fibrosis were shown to be associated with high levels of TNF- α (90, 121, 122).

The ameliorating effect of nematode coinfection on the severity of schistosomiasis is similar to that exerted on a variety of autoimmune diseases (9–15, 123), thus offering a collective explanation for the lower incidence of these T-cell-mediated conditions in areas where helminths are endemic. Such an effect of nematodes with relatively little intrinsic pathogenicity appears to be beneficial for the host and is currently being explored as a therapeutic means to control inflammatory bowel disease in humans (124) and possibly other autoimmune diseases (10). On the other hand, the helminths may be detrimental under conditions in which a strong proinflammatory response is necessary to control other infectious agents (20, 125–128).

In summary, pre-exposure to intestinal nematodes effectively protected mice from severe schistosomiasis by downregulation of pathogenic Th1- and Th17-cell-mediated responses. Regardless, a concept supported by our findings is that, as a whole, natural or therapeutic helminth infections can be important elements in the prevention and amelioration of aberrant or excessive CD4 T-cell-mediated disease.

### 4.1 Study limitations and recommendations

Chronic schistosomiasis is a serious health concern affecting large population worldwide. The present study investigated the effect of co-infection with *T. spiralis* on *S. mansoni* induced liver pathology. Pre-infection with *T. spiralis* showed a promising protective effect against *S. mansoni* liver fibrosis. Further studies are needed to exactly identify the underlying molecular and immunological basis of either parasitic co-infection or parasitic antigens in the protection against chronic schistosomiasis.

## Data Availability

The original contributions presented in the study are included in the article/Supplementary material, further inquiries can be directed to the corresponding authors.

## References

[1] Aguilar-Marcelino L Bautista-Garfias C Zaheer T Maqsood A Salman S Bamarni I. Potential of anisakiasis in foodborne zoonosis. Pakistan Veter J. (2022) 42:80. 10.29261/pakvetj/2022.080

[2] Mahmood Q Younus M Sadiq S Iqbal S Idrees A Khan S. Prevalence and associated risk factors of cystic echinococcosis in food animals–a neglected and prevailing zoonosis. Pak Vet J. (2022) 42:59–64. 10.29261/pakvetj/2022.008

[3] Finlay CM Stefanska AM Walsh KP Kelly PJ Boon L Lavelle EC. Helminth products protect against autoimmunity via innate type 2 cytokines Il-5 and Il-33, which promote eosinophilia. J Immunol. (2016) 196:703–14. 10.4049/jimmunol.1501820 26673140

[4] Capron A Dombrowicz D Capron M. Helminth infections and allergic diseases: from the th2 paradigm to regulatory networks. Clin Rev Allergy Immunol. (2004) 26:25–34. 10.1385/CRIAI:26:1:25 14755073

[5] Dunne DW Cooke A. A worm's eye view of the immune system: consequences for evolution of human autoimmune disease. Nat Rev Immunol. (2005) 5:420–6. 10.1038/nri1601 15864275

[6] Maizels RM. Infections and allergy - helminths, hygiene and host immune regulation. Curr Opin Immunol. (2005) 17:656–61. 10.1016/j.coi.2005.09.001 16202576

[7] Yazdanbakhsh M Kremsner PG van Ree R. Allergy, parasites, and the hygiene hypothesis. Science (New York, NY). (2002) 296:490–4. 10.1126/science.296.5567.490 11964470

[8] Yazdanbakhsh M Matricardi PM. Parasites and the hygiene hypothesis: regulating the immune system? Clin Rev Allergy Immunol. (2004) 26:15–24. 10.1385/CRIAI:26:1:15 14755072

[9] Cooke A Tonks P Jones FM O'Shea H Hutchings P Fulford AJ. Infection with *Schistosoma mansoni* prevents insulin dependent diabetes mellitus in non-obese diabetic mice. Parasite Immunol. (1999) 21:169–76. 10.1046/j.1365-3024.1999.00213.x 10320614

[10] La Flamme AC Canagasabey K Harvie M Bäckström BT. Schistosomiasis protects against multiple sclerosis. Mem Inst Oswaldo Cruz. (2004) 99:33–6. 10.1590/S0074-02762004000900006 15486632

[11] Sewell D Qing Z Reinke E Elliot D Weinstock J Sandor M. Immunomodulation of experimental autoimmune encephalomyelitis by helminth ova immunization. Int Immunol. (2003) 15:59–69. 10.1093/intimm/dxg012 12502726

[12] Zaccone P Fehérvári Z Jones FM Sidobre S Kronenberg M Dunne DW. *Schistosoma mansoni* antigens modulate the activity of the innate immune response and prevent onset of type 1 diabetes. Eur J Immunol. (2003) 33:1439–49. 10.1002/eji.200323910 12731071

[13] Saunders KA Raine T Cooke A Lawrence CE. Inhibition of autoimmune type 1 diabetes by gastrointestinal helminth infection. Infect Immun. (2007) 75:397–407. 10.1128/IAI.00664-06 17043101 PMC1828378

[14] Kitagaki K Businga TR Racila D Elliott DE Weinstock JV Kline JN. Intestinal helminths protect in a murine model of asthma. J immunology. (2006) 177:1628–35. 10.4049/jimmunol.177.3.1628 16849471

[15] Nagayama Y Watanabe K Niwa M McLachlan SM Rapoport B. *Schistosoma mansoni* and alpha-galactosylceramide: prophylactic effect of th1 immune suppression in a mouse model of graves' hyperthyroidism. J immunology. (2004) 173:2167–73. 10.4049/jimmunol.173.3.2167 15265954

[16] Elias D Britton S Kassu A Akuffo H. Chronic helminth infections may negatively influence immunity against tuberculosis and other diseases of public health importance. Expert Rev Anti Infect Ther. (2007) 5:475–84. 10.1586/14787210.5.3.475 17547511

[17] Nacher M Singhasivanon P Yimsamran S Manibunyong W Thanyavanich N Wuthisen R. Intestinal helminth infections are associated with increased incidence of *Plasmodium falciparum* malaria in Thailand. J Parasitol. (2002) 88:55–8. 10.1645/0022-3395(2002)088[0055:IHIAAW]2.0.CO;2 12053980

[18] Noland GS Urban JF Fried B Kumar N. Counter-regulatory anti-parasite cytokine responses during concurrent *Plasmodium yoelii* and intestinal helminth infections in mice. Exp Parasitol. (2008) 119:272–8. 10.1016/j.exppara.2008.02.009 18396282 PMC2441905

[19] Su Z Segura M Morgan K Loredo-Osti JC Stevenson MM. Impairment of protective immunity to blood-stage malaria by concurrent nematode infection. Infect Immun. (2005) 73:3531–9. 10.1128/IAI.73.6.3531-3539.2005 15908382 PMC1111846

[20] Talaat KR Bonawitz RE Domenech P Nutman TB. Preexposure to live *Brugia malayi* microfilariae alters the innate response of human dendritic cells to *Mycobacterium tuberculosis*. J Infect Dis. (2006) 193:196–204. 10.1086/498912 16362883

[21] Tristão-Sá R Ribeiro-Rodrigues R Johnson LT Pereira FE Dietze R. Intestinal nematodes and pulmonary tuberculosis. Rev Soc Bras Med Trop. (2002) 35:533–5. 10.1590/S0037-86822002000500020 12621678

[22] Weng M Huntley D Huang IF Foye-Jackson O Wang L Sarkissian A. Alternatively activated macrophages in intestinal helminth infection: effects on concurrent bacterial colitis. J Immunol. (2007) 179:4721–31. 10.4049/jimmunol.179.7.4721 17878371 PMC3208515

[23] Anthony RM Rutitzky LI Urban JF Stadecker MJ. Gause WC. Protective immune mechanisms in helminth infection. Nat Rev Immunol. (2007) 7:975–87. 10.1038/nri2199 18007680 PMC2258092

[24] El-Derbawy MM Salem HS Raboo M Baiuomy IR Fadil SA Fadil HA. In vivo evaluation of the anti-schistosomal potential of ginger-loaded chitosan nanoparticles on *Schistosoma mansoni*: histopathological, ultrastructural, and immunological changes. Life. (2022) 12:1834. 10.3390/life12111834 36362992 PMC9696985

[25] Nelwan ML. Schistosomiasis: life cycle, diagnosis, and control. Curr Ther Res Clin Exp. (2019) 91:5–9. 10.1016/j.curtheres.2019.06.001 31372189 PMC6658823

[26] Salawu OT Odaibo AB. Maternal schistosomiasis: a growing concern in sub-Saharan Africa. Pathog Glob Health. (2014) 108:263–70. 10.1179/2047773214Y.0000000150 25223633 PMC4216748

[27] Mbugi NO Laizer H Chacha M Mbega E. Prevalence of human schistosomiasis in various regions of *Tanzania* mainland and *Zanzibar*: a systematic review and meta-analysis of studies conducted for the past ten Years (2013-2023). PLoS Negl Trop Dis. (2024) 18:e0012462. 10.1371/journal.pntd.0012462 39250468 PMC11412511

[28] Dag O Erez M Kozan E Mine A Irem I. In vitro anthelmintic activity of five different *Artemisia l. species growing in Türkiye*. Pakistan Veter J. (2023) 2023:2074–7764. 10.29261/pakvetj/2023.087 37915450 PMC10618014

[29] Fadladdin YAJ. Evaluation of antischistosomal activities of crude aqueous extracts of *Artemisia annua, Nigella sativa*, and *Allium sativum* against *Schistosoma mansoni* in hamsters. Biomed Res Int. (2022) 2022:5172287. 10.1155/2022/5172287 35313628 PMC8934242

[30] Hay SI Abajobir AA Abate KH Abbafati C Abbas KM Abd-Allah F. Global, regional, and national disability-adjusted life-years (dalys) for 333 diseases and injuries and healthy life expectancy (hale) for 195 countries and territories, 1990-2016: a systematic analysis for the global burden of disease study 2016. Lancet. (2017) 390:1260–344. 10.1016/S0140-6736(17)32130-X 28919118 PMC5605707

[31] Kahisay M Birhanie M Derso A. Prevalence and intensity of *Schistosoma mansoni* infection and its associated risk factors among patients with and without HIV at chuahit health center, Dembia district, Northwest Ethiopia. Res Rep Trop Med. (2021) 12:25–32. 10.2147/RRTM.S292899 33623470 PMC7896777

[32] Egbuna C Akram M Ifemeje JC. Neglected Tropical Diseases and Phytochemicals in Drug Discovery. New York: Wiley Online Library (2021). 10.1002/9781119617143

[33] Bekana T Abebe E Mekonnen Z Tulu B Ponpetch K Liang S. Parasitological and malacological surveys to identify transmission sites for *Schistosoma mansoni* in Gomma district, south-western Ethiopia. Sci Rep. (2022) 12:17063. 10.1038/s41598-022-21641-2 36224348 PMC9556602

[34] Cheever AW. Differential regulation of granuloma size and hepatic fibrosis in schistosome infections. Memórias do Instituto Oswaldo Cruz. (1997) 92:689–92. 10.1590/S0074-02761997000500024 9566240

[35] Gryseels B Polman K Clerinx J Kestens L. Human schistosomiasis. Lancet. (2006) 368:1106–18. 10.1016/S0140-6736(06)69440-3 16997665

[36] Arnaud V Li J Wang Y Fu X Mengzhi S Luo X. Regulatory role of interleukin-10 and interferon-γ in severe hepatic central and peripheral fibrosis in humans infected with *Schistosoma japonicum*. J Infect Dis. (2008) 198:418–26. 10.1086/588826 18582197 PMC2753300

[37] Takemura Y Kikuchi S Inaba Y. Epidemiologic study of the relationship between schistosomiasis due to *Schistosoma japonicum* and liver cancer/cirrhosis. Am J Trop Med Hyg. (1998) 59:551–6. 10.4269/ajtmh.1998.59.551 9790429

[38] Kandeel M Rehman T Akhtar T Zaheer T Andrabi S Ashraf U. Anti-parasitic applications of nanoparticles: a review. Pak Vet J. (2022) 42:2074–7764. 10.29261/pakvetj/2022.040

[39] Rehman T Elsaid F Magdalena M Toledo G Gentile A Ahmed Gul R. Fasciolosis: recent update in vaccines development and their efficacy. Pak Vet J. (2023) 43:2074–7764. 10.29261/pakvetj/2023.034

[40] Hammouda NA el-Nassery SF Bakr ME el-Gebaly WM Hassan AM. Effect of toxoplasmosis on experimental schistosomiasis. J Egypt Soc Parasitol. (1994) 24:395–406. 8077759

[41] Hammouda NA el-Nassery SF Bakr ME el-Gebali WM abo el-Nazar SY Hassan AM. Immunological and histopathological studies on the effect of toxoplasmosis in experimental schistosomiasis. J Egypt Soc Parasitol. (1994) 24:429–37. 8077762

[42] Sirag SB Christensen NO Frandsen F Monrad J Nansen P. Homologous and heterologous resistance in echinostoma revolutum infections in mice. Parasitology. (1980) 80:479–86. 10.1017/S0031182000000949 7393619

[43] Bazzone LE Smith PM Rutitzky LI Shainheit MG Urban JF Setiawan T. Coinfection with the intestinal nematode heligmosomoides polygyrus markedly reduces hepatic egg-induced immunopathology and proinflammatory cytokines in mouse models of severe schistosomiasis. Infect Immun. (2008) 76:5164–72. 10.1128/IAI.00673-08 18710859 PMC2573333

[44] Liao C Cheng X Liu M Wang X Boireau P. *Trichinella spiralis* and tumors: cause, coincidence or treatment? Anticancer Agents Med Chem. (2018) 18:1091–9. 10.2174/1871520617666171121115847 29173187 PMC6340159

[45] Gottstein B Pozio E Nöckler K. Epidemiology, diagnosis, treatment, and control of trichinellosis. Clin Microbiol Rev. (2009) 22:127–45. 10.1128/CMR.00026-08 19136437 PMC2620635

[46] Bruschi F Dupouy-Camet J. Helminth Infections and Their Impact on Global Public Health. Cham: Springer. (2014). 10.1007/978-3-7091-1782-8

[47] Gao F Wang R Liu M. *Trichinella spiralis*, potential model nematode for epigenetics and its implication in metazoan parasitism. Front Physiol. (2014) 4:410. 10.3389/fphys.2013.00410 24454291 PMC3887316

[48] Malone CJ Oksanen A Mukaratirwa S Sharma R Jenkins E. From wildlife to humans: the global distribution of *Trichinella* species and genotypes in wildlife and wildlife-associated human trichinellosis. Int J Parasitol. (2024) 24:100934. 10.1016/j.ijppaw.2024.100934 38651034 PMC11033181

[49] Ilic N Gruden-Movsesijan A Sofronic-Milosavljevic L. *Trichinella spiralis*: shaping the immune response. Immunol Res. (2012) 52:111–9. 10.1007/s12026-012-8287-5 22392054

[50] Bruschi F Gómez-Morales M. Immune response to parasitic infections-immunity to helminths and novel therapeutic approaches. Clin Infect Dis. (2014) 60:1734. 10.1093/cid/civ175

[51] Park HK Cho MK Choi SH Kim YS Yu HS. *Trichinella spiralis*: infection reduces airway allergic inflammation in mice. Exp Parasitol. (2011) 127:539–44. 10.1016/j.exppara.2010.10.004 21044628

[52] Ashour DS. *Trichinella spiralis* immunomodulation: an interactive multifactorial process. Expert Rev Clin Immunol. (2013) 9:669–75. 10.1586/1744666X.2013.811187 23899237

[53] Wang XL Fu BQ Yang SJ Wu XP Cui GZ Liu MF. *Trichinella spiralis*–a potential anti-tumor agent. Vet Parasitol. (2009) 159:249–52. 10.1016/j.vetpar.2008.10.052 19041180

[54] Molinari JA Ebersole JL. Antineoplastic effects of long-term *Trichinella spiralis* infection on b-16 melanoma. Int Arch Allergy Appl Immunol. (1977) 55:444–8. 10.1159/000231956 591108

[55] Pocock D Meerovitch E. The anti-neoplastic effect of trichinellosis in a syngeneic murine model. Parasitology. (1982) 84:463–73. 10.1017/S0031182000052768 7099711

[56] Luo J Yu L Xie G Li D Su M Zhao X. Study on the mitochondrial apoptosis pathways of small cell lung cancer H446 cells induced by *Trichinella spiralis* muscle larvae ESPS. Parasitology. (2017) 144:793–800. 10.1017/S0031182016002535 28073393

[57] Gruden-Movsesijan A Radulović N Mostarica Stojkovic M Stosic-Grujicic S Milic M Sofronic-Milosavljevic L. *Trichinella spiralis*: modulation of experimental autoimmune encephalomyelitis in da rats. Exp Parasitol. (2008) 118:641–7. 10.1016/j.exppara.2007.12.003 18226814

[58] Gruden-Movsesijan A Ilic N Mostarica-Stojkovic M Stosic-Grujicic S Milic M Sofronic-Milosavljevic L. Mechanisms of modulation of experimental autoimmune encephalomyelitis by chronic *Trichinella spiralis* infection in dark agouti rats. Parasite Immunol. (2010) 32:450–9. 10.1111/j.1365-3024.2010.01207.x 20500676

[59] Du L Tang H Ma Z Xu J Gao W Chen J. The protective effect of the recombinant 53-kda protein of *Trichinella spiralis* on experimental colitis in mice. Dig Dis Sci. (2011) 56:2810–7. 10.1007/s10620-011-1689-8 21476030

[60] Eissa MM Mostafa DK Ghazy AA El Azzouni MZ Boulos LM Younis LK. Anti-arthritic activity of *Schistosoma mansoni* and *Trichinella spiralis* derived-antigens in adjuvant arthritis in rats: role of Foxp3+ Treg Cells. PLoS ONE. (2016) 11:e0165916. 10.1371/journal.pone.0165916 27802332 PMC5089557

[61] Sun S Li H Yuan Y Wang L He W Xie H. Preventive and therapeutic effects of *Trichinella spiralis* adult extracts on allergic inflammation in an experimental asthma mouse model. Parasit Vectors. (2019) 12:326. 10.1186/s13071-019-3561-1 31253164 PMC6599242

[62] Sofronic-Milosavljevic L Ilic N Pinelli E Gruden-Movsesijan A. Secretory products of *Trichinella spiralis* muscle larvae and immunomodulation: implication for autoimmune diseases, allergies, and malignancies. J Immunol Res. (2015) 2015:523875. 10.1155/2015/523875 26114122 PMC4465845

[63] Osborne LC Monticelli LA Nice TJ Sutherland TE Siracusa MC Hepworth MR. Coinfection virus-helminth coinfection reveals a microbiota-independent mechanism of immunomodulation. Science (New York, NY). (2014) 345:578–82. 10.1126/science.1256942 25082704 PMC4548887

[64] Maizels RM Gause WC. Immunology. How helminths go viral. Science (New York, NY). (2014) 345:517–8. 10.1126/science.1258443 25082688

[65] Gamble HR. Detection of trichinellosis in pigs by artificial digestion and enzyme immunoassay. J Food Prot. (1996) 59:295–8. 10.4315/0362-028X-59.3.295 10463449

[66] Abd El Wahab WM El-Badry AA Mahmoud SS El-Badry YA El-Badry MA Hamdy DA. Ginger (*Zingiber officinale*)-derived nanoparticles in *Schistosoma mansoni* infected mice: hepatoprotective and enhancer of etiological treatment. PLoS Negl Trop Dis. (2021) 15:e0009423. 10.1371/journal.pntd.0009423 34014936 PMC8171924

[67] Murambiwa P Silas E Mdleleni Y Mukaratirwa S. Chemokine, cytokine and haematological profiles in sprague-dawley rats co-infected with *Plasmodium berghei* anka and *Trichinella zimbabwensis*-a laboratory animal model for malaria and tissue-dwelling nematodes co-infection. Heliyon. (2020) 6:e03475. 10.1016/j.heliyon.2020.e03475 32140591 PMC7044667

[68] Duvall RH DsWitt W. An improved perfusion technique for recovering adult schistosomes from laboratory animals. Am J Trop Med Hyg. (1967) 16:483–6. 10.4269/ajtmh.1967.16.483 4952149

[69] Pellegrino J Oliveira CA Faria J Cunha AS. New approach to the screening of drugs in experimental schistosomiasis mansoni in mice. Am J Trop Med Hyg. (1962) 11:201–15. 10.4269/ajtmh.1962.11.201 14484966

[70] Kloetzel K. A suggestion for the prevention of severe clinical forms of schistosomiasis mansoni. Bull World Health Organ. (1967) 37:686. 5301745 PMC2554355

[71] Younis S Diab R Eltarahony M Arafa F. The anti-schistosomal activity of magnetite and zero-valent iron nanoparticles on *Schistosoma mansoni*: an in vivo study. Parasitologists United J. (2021) 14:1126. 10.21608/puj.2021.88219.1126

[72] Kiernan J. Histological and Histochemical Methods, Theory and Practice. Delhi, India: Butter Worth Heinemann Replika Press Pvt Ltd. (1999).

[73] Zaalouk T Abu-Sheishaa G Aly I. Regulation of liver fibrosis during murine schistosomiasis mansoni. Egypt J Hospital Med. (2020) 81:1275. 10.21608/ejhm.2020.112318

[74] Pearce EJ MacDonald AS. The immunobiology of schistosomiasis. Nat Rev Immunol. (2002) 2:499–511. 10.1038/nri843 12094224

[75] Cortez DM Feldman MD Mummidi S Valente AJ Steffensen B Vincenti M. Il-17 stimulates mmp-1 expression in primary human cardiac fibroblasts via P38 Mapk- and Erk1/2-Dependent C/Ebp-Beta, Nf-Kappab, and Ap-1 activation. Am J Physiol Heart Circ Physiol. (2007) 293:H3356–65. 10.1152/ajpheart.00928.2007 17921324

[76] Lemmers A Moreno C Gustot T Maréchal R Degré D Demetter P. The interleukin-17 pathway is involved in human alcoholic liver disease. Hepatology. (2009) 49:646–57. 10.1002/hep.22680 19177575

[77] Harrington LE Hatton RD Mangan PR Turner H Murphy TL Murphy KM. Interleukin 17-producing Cd4+ effector T cells develop via a lineage distinct from the t helper type 1 and 2 lineages. Nat Immunol. (2005) 6:1123–32. 10.1038/ni1254 16200070

[78] Yingchun L Huihan W Rong Z Guojun Z Ying Y Zhuogang L. Antitumor activity of asiaticoside against multiple myeloma drug-resistant cancer cells is mediated by autophagy induction, activation of effector caspases, and inhibition of cell migration, invasion, and stat-3 signaling pathway. Med Sci Monitor. (2019) 25:1355. 10.12659/MSM.913397 30785126 PMC6391856

[79] Liu S An J Qi F Yang L Tian Z Zhao M. Neuroprotective effects of asiaticoside. Neural Regen Res. (2014) 9:1275–82. 10.4103/1673-5374.137574 25221579 PMC4160853

[80] Koyama Y Brenner DA. Liver inflammation and fibrosis. J Clin Invest. (2017) 3:55–64. 10.1172/JCI88881 28045404 PMC5199698

[81] Jones SA Jenkins BJ. Recent insights into targeting the IL-6 cytokine family in inflammatory diseases and cancer. Nat Rev Immunol. (2018) 18:773–89. 10.1038/s41577-018-0066-7 30254251

[82] Ghasemi H. Roles of IL-6 in ocular inflammation: a review. Ocu. Immunol Inflam. (2018) 26:37–50. 10.1080/09273948.2016.1277247 28146368

[83] Tseng Y-J Dong L Liu Y-F Xu N Ma W Weng S-Q. Role of autophagy in chronic liver inflammation and fibrosis. Curr Protein Peptide Sci. (2019) 20:817–22. 10.2174/1389203720666190305165203 30843487

[84] Wang J Li Z Wang Z Yu Y Li D Li B. Nanomaterials for combinational radio–immuno oncotherapy. Adv Funct Mater. (2020) 30:1910676. 10.1002/adfm.201910676

[85] Yu L He P Xu Y Kou X Yu Z Xie X. Manipulations of DNA four-way junction architecture and DNA modified Fe3O4@ Au nanomaterials for the detection of miRNA. Sensors Actuat B. (2020) 313:128015. 10.1016/j.snb.2020.128015

[86] Larkin BM Smith PM Ponichtera HE Shainheit MG Rutitzky LI Stadecker MJ. Induction and regulation of pathogenic Th17 cell responses in schistosomiasis. In: Seminars in immunopathology. Springer (2012). 10.1007/s00281-012-0341-9 PMC3690599 23096253

[87] Duffield JS Lupher M Thannickal VJ. Wynn T. Host responses in tissue repair and fibrosis. Ann Rev Pathol. (2013) 8:241–76. 10.1146/annurev-pathol-020712-163930 23092186 PMC3789589

[88] El-Sayed NM Fathy GM Abdel-Rahman SA El-Shafei MA. Cytokine patterns in experimental schistosomiasis mansoni infected mice treated with silymarin. J Parasitic Dis. (2016) 40:922–9. 10.1007/s12639-014-0606-4 27605811 PMC4996219

[89] Kitani A Fuss I Nakamura K Kumaki F Usui T Strober W. Transforming growth factor (Tgf)-beta1-producing regulatory T cells induce smad-mediated interleukin 10 secretion that facilitates coordinated immunoregulatory activity and amelioration of Tgf-beta1-mediated fibrosis. J Exp Med. (2003) 198:1179–88. 10.1084/jem.20030917 14557415 PMC2194234

[90] Henri S Chevillard C Mergani A Paris P Gaudart J Camilla C. Cytokine regulation of periportal fibrosis in humans infected with *Schistosoma mansoni*: Ifn-gamma is associated with protection against fibrosis and Tnf-alpha with aggravation of disease. J Immunol. (2002) 169:929–36. 10.4049/jimmunol.169.2.929 12097398

[91] Zinn-Justin A Marquet S Hillaire D Dessein A Abel L. Genome search for additional human loci controlling infection levels by *Schistosoma mansoni*. Am J Trop Med Hyg. (2001) 65:754–8. 10.4269/ajtmh.2001.65.754 11791970

[92] Rutitzky LI Bazzone L Shainheit MG Joyce-Shaikh B Cua DJ Stadecker MJ. Il-23 is required for the development of severe egg-induced immunopathology in schistosomiasis and for lesional expression of Il-171. J Immunol. (2008) 180:2486–95. 10.4049/jimmunol.180.4.2486 18250458

[93] Rutitzky LI Hernandez HJ Stadecker MJ. Th1-polarizing immunization with egg antigens correlates with severe exacerbation of immunopathology and death in schistosome infection. Proc Natl Acad Sci U S A. (2001) 98:13243–8. 10.1073/pnas.231258498 11606762 PMC60855

[94] Rutitzky LI Lopes da. Rosa JR, Stadecker MJ. Severe Cd4 T cell-mediated immunopathology in murine schistosomiasis is dependent on Il-12p40 and correlates with high levels of Il-17. J Immunol. (2005) 175:3920–6. 10.4049/jimmunol.175.6.3920 16148138

[95] Qiu W Guo K Yi L Gong Y Huang L Zhong W. Resolvin E1 reduces hepatic fibrosis in mice with *Schistosoma japonicum* infection. Exp Ther Med. (2014) 7:1481–5. 10.3892/etm.2014.1641 24926330 PMC4043616

[96] Kastelein RA Hunter CA Cua DJ. Discovery and biology of Il-23 and Il-27: related but functionally distinct regulators of inflammation. Annu Rev Immunol. (2007) 25:221–42. 10.1146/annurev.immunol.22.012703.104758 17291186

[97] Park H Li Z Yang XO Chang SH Nurieva R Wang YH. A distinct lineage of CD4 t cells regulates tissue inflammation by producing interleukin 17. Nat Immunol. (2005) 6:1133–41. 10.1038/ni1261 16200068 PMC1618871

[98] Aggarwal S Ghilardi N Xie MH de Sauvage FJ Gurney AL. Interleukin-23 promotes a distinct CD4 T cell activation state characterized by the production of interleukin-17. J Biol Chem. (2003) 278:1910–4. 10.1074/jbc.M207577200 12417590

[99] Bettelli E Carrier Y Gao W Korn T Strom TB Oukka M. Reciprocal developmental pathways for the generation of pathogenic effector Th17 and regulatory T cells. Nature. (2006) 441:235–8. 10.1038/nature04753 16648838

[100] Korn T Bettelli E Gao W Awasthi A Jäger A Strom TB. Il-21 initiates an alternative pathway to induce proinflammatory T(H)17 cells. Nature. (2007) 448:484–7. 10.1038/nature05970 17581588 PMC3805028

[101] Langrish CL Chen Y Blumenschein WM Mattson J Basham B Sedgwick JD. Il-23 drives a pathogenic T cell population that induces autoimmune inflammation. J Exp Med. (2005) 201:233–40. 10.1084/jem.20041257 15657292 PMC2212798

[102] Sutton C Brereton C Keogh B Mills KH Lavelle EC. A crucial role for interleukin (Il)-1 in the induction of Il-17-producing t cells that mediate autoimmune encephalomyelitis. J Exp Med. (2006) 203:1685–91. 10.1084/jem.20060285 16818675 PMC2118338

[103] Veldhoen M Hocking RJ Atkins CJ Locksley RM Stockinger B. Tgfbeta in the context of an inflammatory cytokine milieu supports de novo differentiation of il-17-producing T cells. Immunity. (2006) 24:179–89. 10.1016/j.immuni.2006.01.001 16473830

[104] Wang B Liang S Wang Y Zhu XQ Gong W Zhang HQ. Th17 down-regulation is involved in reduced progression of schistosomiasis fibrosis in ICOSL KO mice. PLoS Negl Trop Dis. (2015) 9:e0003434. 10.1371/journal.pntd.0003434 25590646 PMC4295877

[105] Franco KGS de Amorim FJR Santos MA Rollemberg CVV de Oliveira FA França AVC. Association of Il-9, Il-10, and Il-17 cytokines with hepatic fibrosis in human *Schistosoma mansoni* infection. Front Immunol. (2021) 12:779534. 10.3389/fimmu.2021.779534 34970264 PMC8712476

[106] Shainheit MG Lasocki KW Finger E Larkin BM Smith PM Sharpe AH. The pathogenic th17 cell response to major schistosome egg antigen is sequentially dependent on il-23 and Il-1β. J immunology. (2011) 187:5328–35. 10.4049/jimmunol.1101445 22003203 PMC3653625

[107] Kalantari P Morales Y Miller EA Jaramillo LD Ponichtera HE Wuethrich MA. Cd209a synergizes with dectin-2 and mincle to drive severe th17 cell-mediated schistosome egg-induced immunopathology. Cell Rep. (2018) 22:1288–300. 10.1016/j.celrep.2018.01.001 29386115 PMC5815841

[108] Meng F Wang K Aoyama T Grivennikov SI Paik Y Scholten D. Interleukin-17 signaling in inflammatory, kupffer cells, and hepatic stellate cells exacerbates liver fibrosis in mice. Gastroenterology. (2012) 143:765–76.e3. 10.1053/j.gastro.2012.05.049 22687286 PMC3635475

[109] Ramani K Biswas PS. Interleukin-17: friend or foe in organ fibrosis. Cytokine. (2019) 120:282–8. 10.1016/j.cyto.2018.11.003 30772195 PMC6555688

[110] Shainheit MG Smith PM Bazzone LE Wang AC Rutitzky LI Stadecker MJ. Dendritic cell Il-23 and Il-1 production in response to schistosome eggs induces th17 cells in a mouse strain prone to severe immunopathology. J Immunol. (2008) 181:8559–67. 10.4049/jimmunol.181.12.8559 19050275 PMC2663362

[111] do Nascimento WR Nóbrega CG Fernandes ED Santos PD Melo FL Albuquerque MC. *Schistosoma mansoni* infection decreases il-33-mrna expression and increases cxcl9 and cxcl10 production by peripheral blood cells. Med Microbiol Immunol. (2022) 211:211–8. 10.1007/s00430-022-00745-6 35819523

[112] Koch DT Koliogiannis D Drefs M Schirren M von Ehrlich-Treuenstätt V Nieß H. Baseline interleukin-6 as a preoperative biomarker for liver fibrosis. Visceral Med. (2024) 39:184–92. 10.1159/000535627 38205272 PMC10775852

[113] Li Y Zhao J Yin Y Li K Zhang C Zheng Y. The role of il-6 in fibrotic diseases: molecular and cellular mechanisms. Int J Biol Sci. (2022) 18:5405–14. 10.7150/ijbs.75876 36147459 PMC9461670

[114] Seki E De Minicis S Osterreicher CH Kluwe J Osawa Y Brenner DA. Tlr4 enhances Tgf-beta signaling and hepatic fibrosis. Nat Med. (2007) 13:1324–32. 10.1038/nm1663 17952090

[115] Dewidar B Meyer C Dooley S Meindl-Beinker AN. TGF-β in hepatic stellate cell activation and liver fibrogenesis-updated 2019. Cells. (2019) 8:1419. 10.3390/cells8111419 31718044 PMC6912224

[116] Li MO Wan YY Sanjabi S Robertson A-KL Flavell RA. Transforming growth factor-β regulation of immune responses. Annu Rev Immunol. (2006) 24:99–146. 10.1146/annurev.immunol.24.021605.090737 16551245

[117] Tallima H Salah M Guirguis FR El Ridi R. Transforming growth factor-β and th17 responses in resistance to primary murine schistosomiasis mansoni. Cytokine. (2009) 48:239–45. 10.1016/j.cyto.2009.07.581 19717308

[118] Gulen MF Kang Z Bulek K Youzhong W Kim TW Chen Y. The receptor Sigirr suppresses Th17 cell proliferation via inhibition of the interleukin-1 receptor pathway and MTOR kinase activation. Immunity. (2010) 32:54–66. 10.1016/j.immuni.2009.12.003 20060329 PMC3015141

[119] Gieling RG Wallace K Han YP. Interleukin-1 participates in the progression from liver injury to fibrosis. Am J Physiol Gastroint Liver Physiol. (2009) 296:G1324–31. 10.1152/ajpgi.90564.2008 19342509 PMC2697947

[120] Mancini R Benedetti A Jezequel AM. An interleukin-1 receptor antagonist decreases fibrosis induced by dimethylnitrosamine in rat liver. Virchows Archiv. (1994) 424:25–31. 10.1007/BF00197389 7981900

[121] Booth M Vennervald BJ Butterworth AE Kariuki HC Amaganga C Kimani G. Exposure to malaria affects the regression of hepatosplenomegaly after treatment for *Schistosoma mansoni* infection in kenyan children. BMC Med. (2004) 2:36. 10.1186/1741-7015-2-36 15450118 PMC522803

[122] de Jesus AR Magalhães A Miranda DG Miranda RG Araújo MI de Jesus AA. Association of type 2 cytokines with hepatic fibrosis in human *Schistosoma mansoni* infection. Infect Immun. (2004) 72:3391–7. 10.1128/IAI.72.6.3391-3397.2004 15155645 PMC415716

[123] Elliott DE Setiawan T Metwali A Blum A Urban JF Weinstock JV. Heligmosomoides polygyrus inhibits established colitis in il-10-deficient mice. Eur J Immunol. (2004) 34:2690–8. 10.1002/eji.200324833 15368285

[124] Summers RW Elliott DE Urban JF Thompson R Weinstock JV. *Trichuris suis* therapy in Crohn's disease. Gut. (2005) 54:87–90. 10.1136/gut.2004.041749 15591509 PMC1774382

[125] Dunne DW Riley EM. Immunity, morbidity and immunoepidemiology in parasite infections. Parasite Immunol. (2004) 26:425–8. 10.1111/j.0141-9838.2004.00737.x 15771678

[126] Helmby H Kullberg M Troye-Blomberg M. Altered immune responses in mice with concomitant *Schistosoma mansoni* and *Plasmodium chabaudi* infections. Infect Immun. (1998) 66:5167–74. 10.1128/IAI.66.11.5167-5174.1998 9784518 PMC108644

[127] Khan IA Hakak R Eberle K Sayles P Weiss LM Urban JF. Coinfection with heligmosomoides polygyrus fails to establish Cd8+ T-cell immunity against *Toxoplasma gondii*. Infect Immun. (2008) 76:1305–13. 10.1128/IAI.01236-07 18195022 PMC2258819

[128] Legesse M Erko B Balcha F. Increased parasitaemia and delayed parasite clearance in *Schistosoma mansoni* and *Plasmodium berghei* co-infected mice. Acta Trop. (2004) 91:161–6. 10.1016/j.actatropica.2004.04.002 15234665

