# Supplementary material for: Prior Trichinella spiralis infection protects against Schistosoma mansoni induced hepatic fibrosis
Source: Front Vet Sci. 2024 Oct 8;11:1443267. doi: 10.3389/fvets.2024.1443267 (PMC11494294; doi:10.3389/fvets.2024.1443267)
Supplement: Supplementary file 1 [file Table_1.docx]

**Table 1. *S. mansoni* worm burden in mice infected with only *S. mansoni* compared to mice that were pre- infected with T. spiralis followed by *S. mansoni***

**Worm Type Worm burden**

|  | Mean ± SD | % Total worm  burden reduction | F test | Post hoc |
| --- | --- | --- | --- | --- |
| **Male** |  |  | 3.6 | 0.045 |
| **GII: *Sm*-infected** | 1.86±0.89 |  |  |  |
| **GIII: *T.s* –*Sm*** | 1.17±0.41 | 41 |  |  |
| **Female** |  |  | 18.6 | 0.002* |
| **GII: *Sm-infected*** | 0.71±0.49 |  |  |  |
| **GIII: *T.s* – *Sm*** | 0 | 100 |  |  |
| **Couple** |  |  | 0.112 | 0.744 |
| **GII: *Sm-infected*** | 7.71±0.76 |  |  |  |
| **GIII: *T.s* – *Sm*** | 1±0.63 | 87.1 |  |  |
| **Total** |  |  | 2.9 | 0.062 |
| **GII: *Sm-infected*** | 18±2.16 |  |  |  |
| **GIII: *T.s* – *Sm*** | 3.17±0.98 | 82.4 |  |  |

** indicates significant difference between both studied groups. T.s*–*Sm: mice group infected with T. spiralis then S. mansoni. Sm: mice group infected with S. mansoni only*

**Table 2. *S. mansoni* tissue egg count in mice infected with only *S. mansoni c*ompared to mice that were pre-infected with T. spiralis followed by *S. mansoni***

| **Groups** |  | **Intestinal egg cou** | **nt** |  |
| --- | --- | --- | --- | --- |
|  | Mean ± SD | % Reduction | F test | Post hoc |
| **GII: *Sm-infected*** | 8619.43± 727.52 |  | 9.9 | 0.009* |
| **GIII: *T.s* – *Sm*** | 207.2±64.3 | 98 |  |  |
|  |  | **Liver egg count** |  |  |
|  | Mean ± SD | % Reduction | 8.4 | 0.014* |
| **GII: *Sm-infected*** | 7916.86±771.34 |  |  |  |
| **GIII: *T.s* – *Sm*** | 279±87.2 | 96.5 |  |  |
|  | **Total Tissue egg count** | | | |
|  | Mean ± SD | % Reduction | 12.6 | 0.005* |
| **GII: *Sm-infected*** | 16536.29± 1399.49 |  |  |  |
| **GIII: *T.s* – *Sm*** | 536±161.14 | 96.8 |  |  |

** indicates significant difference between both studied groups. T.s* – *Sm: mice group infected with T. spiralis then S. mansoni. Sm: mice group infected with S. mansoni only*
